# Supplementary material for: Not discussed: Inequalities in narrative text data for suicide deaths in the National Violent Death Reporting System
Source: PLoS One. 2021 Jul 16;16(7):e0254417. doi: 10.1371/journal.pone.0254417 (PMC8284808; doi:10.1371/journal.pone.0254417)
Supplement: S1 Appendix — (DOCX) [file pone.0254417.s001.docx]

## **S1 Appendix: Exploring the “information potential” of short and long narrative texts**

## The NVDRS contains “gateway” variables that, according to the NVDRS Data Analysis Guide for RAD Users, “lock or unlock access to other variables in NVDRS” (p.5). The variable “CircumstancesKnown” is a gateway variable that allows NVDRS abstractors to enter LE and CME circumstances. The value “1,” indicating the presence of circumstances, is assigned to the “CircumstancesKnown” variable whenever an abstractor has evidence of any contributing circumstances to the death. For example, whether the decedent had a history of depression or recently experienced a crisis of some sort, among others. The Data Analysis Guide instructs researchers who are interested in examining salient circumstances to limit their analysis to those observations in which the circumstances are known.

## Accordingly, Analysis 2 (analysis of narrative length) was limited to those observations in which the NVDRS coders indicated that *circumstances were known* via this variable, as the intent of the narrative is to provide a detailed description of these circumstances. This resulted in the exclusion of an additional n=27,317 cases from the analysis. However, as shown by the table below, in many cases (n=19,816 (9% of single suicide or undetermined deaths) for C/ME narratives and n=13,316 (7.1% of single suicide or undetermined deaths) for LE narratives) the narrative was still longer than 30 characters in length.

| Appendix Table 1: Mean narrative length by whether NVDRS coders indicated that circumstances were known | | | |
| --- | --- | --- | --- |
|  | Total N | Circumstances known | |
|  |  | No | Yes |
| C/ME narratives >30 characters | 211046 (95%) | 19816 (9.0%) | 201230 (91.0%) |
| LE narratives >30 characters | 186935 (80%) | 1316 (7.1%) | 173619 (92.9) |
| Median [Q1; Q3] |  |  |  |
| C/ME narratives >30 characters | 492 [317; 737] | 271 [150; 455] | 513 [339; 761] |
| LE narratives >30 characters | 523 [313; 837] | 254 [120; 449] | 546 [335; 864] |

## The Data User’s Guide also states that “If a circumstance is not endorsed by the state data abstractor, this does not mean that the circumstance was not present. It means that state data abstractors cannot make assumptions about circumstances beyond those indicated in the required source documents (i.e., law enforcement report or coroner/medical examiner report). Therefore, results and findings should be evaluated cautiously.”

To explore whether there was any relevant information in the narratives of those decedents who had texts >30 characters but whose circumstances were coded as “unknown,” we examined the content of a random sample of 20 narratives of each type (i.e., 20 of the 19,816 C/ME narratives) and 20 from the 1,316 LE narratives). The information described in these 40 narratives is summarized below, as well as two example narratives which have had several details changed to protect privacy.

| Appendix Table 2: Summary of the contents of a random sample of texts which are >30 characters in length but are coded as “Circumstances Unknown” | | |
| --- | --- | --- |
|  | C/ME narratives (n=20) | LE narratives (n=20) |
| Described demographic characteristic of decedent (e.g., age, race, sex) | 17/20 | 15/20 |
| Described means of injury (e.g., firearm, hanging, poisoning) | 18/20 | 12/20 |
| Described the scene (e.g., location of the deceased, items found at the location) | 14/20 | 14/20 |
| Described health history of the decedent (e.g., history of cancer, history of depression, history of suicide attempt) | 8/20 | 2/20 |
| Described events around the time of death (e.g., witness statements, information regarding recent or ongoing situations) | 5/20 | 5/20 |
| Edited example of a narrative that described events around the time of death | *Victim was found inside of a non-for-profit residential facility. Victim had been staying in the facility while his son was hospitalized nearby. Victim ingested a large quantity of diphenhydramine and two bottles of alcohol-based hand sanitizer. No other information is known at this time.* | *Victim was found on his grandmother’s bedroom with an intentional, self-inflicted gunshot wound. The handgun belonged to the victim’s grandmother and she kept it in her bedroom loaded and unlocked. According to the grandmother, there were no prior attempts, the victim did not have any mental health issues, and the grandmother could not think of any reason why the victim would do this. No information was given regarding the victim being in a depressed mood or any recent threats* |

**Annotated examples of short (31 to <200 characters) and long (>500 characters) narratives**

To explore the notion that longer narratives have more “information potential”, we provide five examples of long (>500 characters) narratives (some C/ME and some LE), alongside annotation that indicates what details, elements, and features are specified in these texts. We note that multiple details of these narratives have been changed to preserve confidentiality.

| **Examples** | **Details/features specified** |
| --- | --- |
| **Short (31 to <200 characters) text** |  |
| Victim is a 52 year old male who was found unresponsive on his bathroom floor with self-inflicted gunshot wound to the head. Weapon recovered scene. No note. Victim’s prescription medications included alprazolam, tramadol, xanax, oxycodone. | - Age and sex of victim. - Manner on injury. - Location of injury. - Prescription medications, indicating history of treatment for anxiety and pain. |
| 35 year old male died of suicide by self-inflicted hanging outside his residence. The victim was found suspended by a tree the day after he had an argument with his mother. The victim had multiple active warrants for his arrest due to failure to pay child support for multiple children. The victim also had a medical history of COPD, ADHD, and smoking. | - Age and sex of victim. - Manner of injury. - Location of injury. - Parent, but estranged from children. - Recent law enforcement involvement. - Acute (<24 hours) upsetting interpersonal event. - Medical and psychiatric history. |
| Officers were dispatched in reference to a person that had hung himself in the garage. Victim was pronounced dead on the scene. Victim was a 35 black male. | - Age, sex and race of victim. - Manner of injury. - Location of injury. |
| 45 year old white male died from a gunshot wound to an unspecified part of the body, inside a motor vehicle parked in another person’s drive way. Manner of death is unspecified. No further details. | - Age, sex and race of victim. - Manner of injury. - Location of injury. |
| 55 year old white male shot himself at his father’s residence. He recently lost his job of 10 years and was in serious financial trouble. | - Age, sex and race of victim. - Manner of injury. - Location of injury. - Recent job loss, financial concerns. |
| **Long (>500 characters) text** |  |
| This 30 year old female victim died as a result of drug toxicity of citalopram, alcohol and hydroxyzine. All medications belonged to the victim, one of which she took for her diagnosed history of depression. The victim had 4 children, none of which lived with her as they had all been removed from the home by social services. The evening prior the victim had been upset over a text message she had received from an ex-boyfriend. The ex-boyfriend had said he was engaged as was going to be married on the same date that he and the victim had broken up. When her mother tried to discuss the text message with the victim she became very angry and hit her mother. This was the last time her mother saw her. Her friend had also been at her residence and said she saw the victim shortly before midnight as she left to go home. She was found the following morning unresponsive in bed. Notes of suicidal intent were written on the wall above her bed, including on her body. | - Age and sex of the victim. - Manner of injury. - Location of injury. - History of depression that was being treated with medication. - Parent, but children had been legally removed from her care. - Acute (<24 hours prior) upsetting interpersonal event. - Recent contact with family/friends. |
| A 58 year old female with intentional, self-inflicted alprazolam poisoning. Victim was found at home. Multiple empty medication bottles were found at the scene. Victim had a history of anxiety disorders and was currently in treatment. There was no mention of the victim being in a depressed mood. Victim was last known alive two and a half weeks prior. No information known about prior attempts. Victim left a note. | - Age and sex of the victim. - Manner of injury. - Location of injury. - History of anxiety that was being treated with medication. - No recent contact with family/friends. |
| A 25 year old man died following the combination use of medication he had been given for a recent back injury. Manner of death is undetermined. Victim had a history of diagnosed depression. Victim had a history of overdosing on prescription drugs and over medicating with alcohol assumption. Victim had attempted suicide the previous year. Previous evening the victim went to a friend’s home to “get drunk.” Found at home. No other circumstances are known. | - Age and sex of the victim. - Manner of injury. - Location on injury. - History of depression, but unclear if being treated. - History of overdose. - History of alcohol misuse. - History of suicide attempt. - Recent contact with friends. |
| A 52 year old male died of suicide by asphyxia due to inhalation of exhaust gases from automobile gasoline engine in a parking lot. Victim was found in a vehicle. A suicide note was found inside the vehicle. The victim had been depressed after life partner passed away several years ago. Victim had also been involved with a situation at work and was having money concerns. | - Age and sex of the victim. - Manner of injury. - Location of injury. - Long-standing bereavement. - Recent concerns at work/finances. |
| Law enforcement responded to victim’s apartment for a welfare check after receiving 911 call reporting victim had made suicidal threats and could not be reached by phone. Upon arrival at the apartment, law enforcement was unable to get a response at the door, entered the unlocked apartment due to nature of call, and discovered victim laying on the floor of her bedroom next to her bed. Law enforcement observed several empty beer cans near victim as well as several empty packets of Z-Quil. Victim was unresponsive and cold to the touch and did not appear to have a pulse. EMS arrived on scene to confirm victim had indeed passed. Law enforcement also found a small amount of marijuana and a pipe. In the victim’s medicine cabinet were several medications, law enforcement collected these along with two large purses containing personal items. Law enforcement also spoke with victim’s live-in boyfriend, who apparently also called 911 earlier that day reporting victim had wanted to harm herself. At that time, the boyfriend had stayed on the phone with law enforcement while he returned to the apartment to check on victim early that morning. He stated he had difficulty opening the apartment door initially as victim had barricaded it. Once inside the apartment the boyfriend found the victim asleep in her bed and shook her to wake her. Victim seemed coherent, so the boyfriend told law enforcement everything was OK and they could check on the victim later. The boyfriend stayed with the victim and they talked for a while before the boyfriend told her he was leaving. The boyfriend said the victim was still upset, as he had just broken up with her, but he thought the victim was going to be alright. The boyfriend left the door unlocked when he left. Law enforcement obtained a warrant to examine the apartment once more and had negative results finding any new evidence that indicated a homicide had occurred. Law enforcement attended the autopsy of victim. The pathologist ruled out any suspicion of homicide, however at the time of this report, the official cause of death could not be determined as the toxicology results are still pending. | - Sex of the victim. - Manner of injury. - Location of injury. - Description of the death scene. - Recent contact with friends/relatives. - Recent upsetting interpersonal event (timing not specified). - Recent suicidal ideation/threats. - Police investigation to rule out homicide. |
