# Supplementary material for: Not discussed: Inequalities in narrative text data for suicide deaths in the National Violent Death Reporting System
Source: PLoS One. 2021 Jul 16;16(7):e0254417. doi: 10.1371/journal.pone.0254417 (PMC8284808; doi:10.1371/journal.pone.0254417)
Supplement: S1 Table — (DOCX) [file pone.0254417.s002.docx]

| **S1 Table. Logistic Regression of Missing Status for NVDRS Narratives Abstracted from Coroner/Medical Examiner and Law Enforcement Reports.** | | | |  |
| --- | --- | --- | --- | --- |
|  | | | |  |
|  | Odd Ratio (95% CI) | | |  |
|  |  | | |  |
|  | CME Narrative | LE Narrative | |  |
|  | | | |  |
| Constant | 0.02^***^ (0.02, 0.03) | 0.12^***^ (0.11, 0.13) | |  |
| **Incident Year** *(ref = 2017)* |  |  |  |  |
| 2003 | 0.91 (0.65, 1.27) | 1.87^***^ (1.65, 2.12) | |  |
| 2004 | 0.86 (0.63, 1.16) | 2.13^***^ (1.90, 2.38) | |  |
| 2005 | 1.32^*^ (0.99, 1.78) | 2.10^***^ (1.87, 2.35) | |  |
| 2006 | 1.66^***^ (1.25, 2.20) | 1.41^***^ (1.26, 1.58) | |  |
| 2007 | 1.30^*^ (0.98, 1.73) | 1.35^***^ (1.20, 1.51) | |  |
| 2008 | 1.45^***^ (1.09, 1.92) | 0.98 (0.87, 1.10) | |  |
| 2009 | 1.95^***^ (1.47, 2.58) | 0.76^***^ (0.68, 0.86) | |  |
| 2010 | 1.46^***^ (1.09, 1.94) | 0.97 (0.87, 1.09) | |  |
| 2011 | 1.44^**^ (1.06, 1.94) | 0.97 (0.86, 1.09) | |  |
| 2012 | 0.84 (0.60, 1.16) | 0.67^***^ (0.59, 0.77) | |  |
| 2013 | 0.55^*^ (0.30, 1.00) | 0.81^**^ (0.67, 0.98) | |  |
| 2014 | 0.22^***^ (0.08, 0.62) | 0.65^***^ (0.48, 0.87) | |  |
| 2015 | 0.73 (0.45, 1.17) | 0.79^**^ (0.64, 0.98) | |  |
| 2016 | 0.63^**^ (0.43, 0.93) | 1.09 (0.94, 1.28) | |  |
| **Age** *(ref=40-49 years)* | |  |  |  |
| ≤18 | 1.07 (0.91, 1.25) | 0.85^***^ (0.79, 0.91) | |  |
| 19-29 | 1.03 (0.94, 1.13) | 0.90^***^ (0.86, 0.93) | |  |
| 30-39 | 1.05 (0.97, 1.14) | 0.95^**^ (0.91, 0.98) | |  |
| 50-59 | 1.05 (0.97, 1.14) | 1.01 (0.97, 1.04) | |  |
| 60-69 | 1.09^*^ (0.99, 1.20) | 1.04 (0.99, 1.09) | |  |
| 70-79 | 1.28^***^ (1.13, 1.44) | 1.07^**^ (1.01, 1.13) | |  |
| ≥80 | 1.15^**^ (0.99, 1.33) | 1.03 (0.96, 1.10) | |  |
| Unknown/Missing | 0.17^***^ (0.10, 0.29) | 0.42^***^ (0.30, 0.57) | |  |
| **Sex** *(ref=Male)* |  |  | |  |
| Female | 1.01 (0.94, 1.08) | 1.12^***^ (1.09, 1.15) | |  |
| Unknown/Missing | 0.52 (0.23, 1.19) | 0.76 (0.46, 1.25) | |  |
| **Race or Ethnicity** *(ref=White)* | |  | |  |
| American Indian/Alaska Native | 2.30^***^ (1.79, 2.95) | 1.65^***^ (1.42, 1.92) | |  |
| Asian/Pacific Islander | 0.77^**^ (0.59, 0.99) | 1.13^*^ (1.02, 1.26) | |  |
| Black or African American | 0.84^***^ (0.76, 0.94) | 1.17^***^ (1.12, 1.23) | |  |
| Hispanic or Latino | 0.96 (0.84, 1.11) | 1.11^**^ (1.04, 1.18) | |  |
| Other/Unspecified | 2.20^**^ (1.18, 4.13) | 1.58^**^ (1.14, 2.18) | |  |
| Two or more races | 0.71^**^ (0.55, 0.92) | 0.79^***^ (0.71, 0.87) | |  |
| Unknown/Missing | 3.59^***^ (2.04, 6.30) | 3.32^***^ (2.36, 4.67) | |  |
| **Homelessness Status** *(ref=No)* | |  | |  |
| Yes | 0.58^***^ (0.39, 0.87) | 0.80^***^ (0.71, 0.89) | |  |
| Unknown/Missing | 18.10^***^ (16.23, 20.18) | 4.25^***^ (3.96, 4.47) | |  |
| **Education Level** *(ref=High School or GED Diploma)* | |  | |  |
| 8th grade or less | 0.95 (0.83, 1.09) | 1.12^**^ (1.05, 1.20) | |  |
| 9-12th grade, no diploma | 0.95 (0.86, 1.05) | 0.94^**^ (0.90, 0.98) | |  |
| Some college, no degree | 0.83^***^ (0.75, 0.92) | 0.94^**^ (0.90, 0.99) | |  |
| Associate's degree | 0.96 (0.84, 1.10) | 0.96 (0.90, 1.03) | |  |
| Bachelor's degree | 0.74^***^ (0.65, 0.85) | 0.93^**^ (0.88, 0.98) | |  |
| Master's degree | 0.81^**^ (0.66, 0.99) | 0.96 (0.87, 1.04) | |  |
| Professional or Doctorate degree | 0.65^**^ (0.48, 0.88) | 0.88^*^ (0.78, 1.00) | |  |
| Unknown/Missing | 0.81^***^ (0.75, 0.89) | 1.21^***^ (1.17, 1.26) | |  |
| **Marital Status** *(ref=Married/In relationship)* | |  | |  |
| Divorced/Separated | 0.91^**^ (0.85, 0.98) | 0.99 (0.96, 1.02) | |  |
| Single/Never Married | 0.86^***^ (0.80, 0.93) | 0.99 (0.95, 1.02) | |  |
| Widowed | 0.94 (0.84, 1.06) | 1.04 (0.99, 1.10) | |  |
| Unknown/Missing | 1.02 (0.75, 1.39) | 0.92 (0.82, 1.04) | |  |
| **Military Status** *(ref=No)* | |  | |  |
| Yes | 1.11^**^ (1.03, 1.19) | 0.97 (0.94, 1.01) | |  |
| Unknown/Missing | 1.27^***^ (1.14, 1.41) | 1.31^***^ (1.25, 1.38) | |  |
| **Autopsy Performed** *(ref=Yes)* | |  | |  |
| No | 1.94^***^ (1.78, 2.11) | 1.05^**^ (1.02, 1.09) | |  |
| Unknown/Missing | 3.89^***^ (2.54, 5.95) | 1.31 (0.99, 1.68) | |  |
| **Place of Death** *(ref=Home)* | |  | |  |
| Hospice or LTC Facility | 1.45^***^ (1.21, 1.74) | 3.70^***^ (3.34, 4.10) | |  |
| Hospital | 1.04 (0.96, 1.13) | 1.91^***^ (1.84, 1.99) | |  |
| Other | 0.92^**^ (0.85, 1.00) | 1.15^***^ (1.11, 1.19) | |  |
| Unknown/Missing | 4.51^***^ (2.64, 7.71) | 2.69^***^ (2.01, 3.61) | |  |
|  | | | |  |
| Observations | 233,108 | 233,108 | |  |
|  | | | |  |
| Note: ^*^p<0.1;^**^p<0.05;^***^p<0.01 | | | | |
