# Supplementary material for: Not discussed: Inequalities in narrative text data for suicide deaths in the National Violent Death Reporting System
Source: PLoS One. 2021 Jul 16;16(7):e0254417. doi: 10.1371/journal.pone.0254417 (PMC8284808; doi:10.1371/journal.pone.0254417)
Supplement: S2 Table — (DOCX) [file pone.0254417.s003.docx]

| **S2 Table. Logistic Regression of Missing Status for NVDRS Narratives Abstracted from Coroner/Medical Examiner and Law Enforcement Reports: Sensitivity Analysis Excluding Sites with <5 Missing Narratives** | | | | |
| --- | --- | --- | --- | --- |
|  | | | | |
|  | OR (95% CI) | | | |
|  |  | | | |
|  | CME Missing | | LE Missing | |
|  | Full | Restricted ǂ | Full | Restricted ǂǂ |
|  | | | | |
| Constant | 0.01^***^ (0.004, 0.01) | 0.01^***^ (0.005, 0.01) | 0.06^***^ (0.06, 0.07) | 0.06^***^ (0.06, 0.07) |
| **Age** *(ref=40-49 years)* |  |  |  |  |
| ≤18 | 0.92 (0.82, 1.05) | 0.92 (0.82, 1.05) | 0.84^***^ (0.79, 0.90) | 0.85^***^ (0.79, 0.90) |
| 19-29 | 1.02 (0.94, 1.10) | 1.02 (0.94, 1.11) | 0.91^***^ (0.87, 0.95) | 0.91^***^ (0.87, 0.95) |
| 30-39 | 1.04 (0.96, 1.11) | 1.03 (0.96, 1.11) | 0.96^**^ (0.92, 1.00) | 0.96^**^ (0.92, 1.00) |
| 50-59 | 1.04 (0.97, 1.11) | 1.03 (0.97, 1.11) | 1.01 (0.97, 1.05) | 1.01 (0.97, 1.05) |
| 60-69 | 1.07 (0.98, 1.16) | 1.07 (0.98, 1.16) | 1.05^**^ (1.00, 1.09) | 1.05^**^ (1.00, 1.09) |
| 70-79 | 1.24^***^ (1.12, 1.36) | 1.24^***^ (1.12, 1.37) | 1.06^*^ (1.00, 1.12) | 1.06^*^ (1.00, 1.12) |
| ≥80 | 1.15^**^ (1.02, 1.30) | 1.14^**^ (1.01, 1.29) | 1.02 (0.95, 1.10) | 1.02 (0.96, 1.10) |
| Unknown/Missing | 0.19^***^ (0.13, 0.28) | 0.18^***^ (0.12, 0.27) | 0.26^***^ (0.19, 0.36) | 0.26^***^ (0.19, 0.36) |
| **Sex** *(ref=Male)* |  |  |  |  |
| Female | 1.02 (0.97, 1.07) | 1.02 (0.96, 1.07) | 1.18^***^ (1.15, 1.22) | 1.18^***^ (1.15, 1.22) |
| Unknown/Missing | 1.54 (0.79, 2.99) | 1.62 (0.83, 3.17) | 2.76^***^ (1.45, 5.24) | 2.75^***^ (1.45, 5.24) |
| **Race or Ethnicity** *(ref=White)* | |  |  |  |
| American Indian/Alaska Native | 3.58^***^ (3.02, 4.25) | 3.58^***^ (3.02, 4.25) | 2.70^***^ (2.42, 3.01) | 2.70^***^ (2.42, 3.01) |
| Asian/Pacific Islander | 0.71^***^ (0.58, 0.87) | 0.71^***^ (0.58, 0.86) | 1.18^***^ (1.08, 1.29) | 1.18^***^ (1.08, 1.29) |
| Black or African American | 0.77^***^ (0.70, 0.84) | 0.77^***^ (0.70, 0.84) | 1.16^***^ (1.11, 1.22) | 1.16^***^ (1.11, 1.22) |
| Hispanic or Latino | 0.87^**^ (0.79, 0.97) | 0.87^**^ (0.78, 0.97) | 1.17^***^ (1.11, 1.24) | 1.17^***^ (1.11, 1.24) |
| Other/Unspecified | 3.74^***^ (2.92, 4.78) | 3.78^***^ (2.95, 4.84) | 1.95^***^ (1.62, 2.33) | 1.95^***^ (1.62, 2.33) |
| Two or more races | 0.69^***^ (0.55, 0.86) | 0.69^***^ (0.55, 0.86) | 0.82^***^ (0.73, 0.92) | 0.82^***^ (0.73, 0.92) |
| Unknown/Missing | 2.14^***^ (1.50, 3.04) | 2.13^***^ (1.49, 3.04) | 2.68^***^ (1.96, 3.66) | 2.67^***^ (1.96, 3.66) |
| **Homelessness Status** *(ref=No)* |  |  |  |  |
| Yes | 0.67^***^ (0.52, 0.88) | 0.67^***^ (0.52, 0.88) | 0.87^**^ (0.78, 0.97) | 0.87^**^ (0.78, 0.97) |
| Unknown/Missing | 18.95^***^ (17.87, 20.08) | 18.99^***^ (17.91, 20.13) | 6.22^***^ (5.93, 6.53) | 6.22^***^ (5.93, 6.53) |
| **Education Level** *(ref=High School or GED Diploma)* | |  |  |  |
| 8th grade or less | 0.91 (0.81, 1.02) | 0.91 (0.81, 1.02) | 1.17^***^ (1.10, 1.25) | 1.17^***^ (1.10, 1.25) |
| 9-12th grade, no diploma | 1.05 (0.97, 1.13) | 1.05 (0.97, 1.13) | 0.92^***^ (0.88, 0.96) | 0.92^***^ (0.88, 0.96) |
| Some college, no degree | 0.88^***^ (0.81, 0.95) | 0.88^***^ (0.81, 0.95) | 0.96^*^ (0.92, 1.01) | 0.97 (0.92, 1.01) |
| Associate's degree | 0.86^***^ (0.78, 0.95) | 0.86^***^ (0.78, 0.95) | 0.99 (0.93, 1.05) | 0.99 (0.93, 1.05) |
| Bachelor's degree | 0.73^***^ (0.67, 0.80) | 0.73^***^ (0.67, 0.80) | 0.90^***^ (0.86, 0.95) | 0.90^***^ (0.86, 0.95) |
| Master's degree | 0.77^***^ (0.67, 0.89) | 0.77^***^ (0.67, 0.89) | 0.95 (0.88, 1.03) | 0.95 (0.88, 1.03) |
| Professional or Doctorate degree | 0.73^***^ (0.59, 0.92) | 0.73^***^ (0.58, 0.91) | 0.84^***^ (0.75, 0.94) | 0.84^***^ (0.74, 0.94) |
| Unknown/Missing | 0.92^**^ (0.85, 1.00) | 0.92^**^ (0.85, 1.00) | 1.49^***^ (1.43, 1.56) | 1.49^***^ (1.43, 1.56) |
| **Marital Status** *(ref=Married/In relationship)* | |  |  |  |
| Divorced/Separated | 0.92^***^ (0.87, 0.97) | 0.92^***^ (0.87, 0.97) | 1.03^*^ (1.00, 1.07) | 1.03^*^ (1.00, 1.06) |
| Single/Never Married | 0.90^***^ (0.85, 0.96) | 0.90^***^ (0.85, 0.96) | 1.01 (0.97, 1.04) | 1.01 (0.97, 1.04) |
| Widowed | 0.95 (0.86, 1.05) | 0.95 (0.86, 1.05) | 1.09^***^ (1.03, 1.15) | 1.08^***^ (1.02, 1.15) |
| Unknown/Missing | 1.20^**^ (1.03, 1.39) | 1.20^**^ (1.03, 1.39) | 1.28^***^ (1.16, 1.41) | 1.28^***^ (1.16, 1.41) |
| **Military Status** *(ref=”No”)* |  |  |  |  |
| Yes | 1.08^**^ (1.02, 1.15) | 1.08^**^ (1.02, 1.15) | 0.99 (0.95, 1.02) | 0.99 (0.95, 1.02) |
| Unknown/Missing | 1.18^***^ (1.08, 1.29) | 1.18^***^ (1.08, 1.29) | 0.84^***^ (0.80, 0.89) | 0.84^***^ (0.80, 0.89) |
| **Autopsy Performed** *(ref”Yes)* |  |  |  |  |
| No | 1.96^***^ (1.87, 2.05) | 1.96^***^ (1.87, 2.05) | 1.01 (0.98, 1.04) | 1.01 (0.98, 1.04) |
| Unknown/Missing | 2.91^***^ (2.46, 3.44) | 2.90^***^ (2.45, 3.43) | 1.56^***^ (1.36, 1.79) | 1.56^***^ (1.36, 1.79) |
| **Place of Death** *(ref=Home)* |  |  |  |  |
| Hospice or LTC Facility | 0.61^***^ (0.51, 0.74) | 0.61^***^ (0.51, 0.74) | 3.60^***^ (3.24, 4.01) | 3.61^***^ (3.24, 4.01) |
| Hospital | 1.05 (0.99, 1.11) | 1.05 (0.99, 1.11) | 2.10^***^ (2.03, 2.16) | 2.10^***^ (2.03, 2.16) |
| Other | 0.89^***^ (0.84, 0.94) | 0.89^***^ (0.84, 0.94) | 1.17^***^ (1.14, 1.21) | 1.17^***^ (1.14, 1.21) |
| Unknown/Missing | 5.38^***^ (4.71, 6.14) | 5.37^***^ (4.70, 6.14) | 1.83^***^ (1.62, 2.06) | 1.82^***^ (1.62, 2.06) |
| **Reporting State** *(ref=Maryland)* |  |  |  |  |
| Alaska | 0.44^***^ (0.27, 0.70) | 0.44^***^ (0.27, 0.70) | 0.07^***^ (0.05, 0.09) | 0.07^***^ (0.05, 0.09) |
| Arizona | 3.81^***^ (3.06, 4.74) | 3.80^***^ (3.06, 4.73) | 3.78^***^ (3.47, 4.12) | 3.78^***^ (3.47, 4.11) |
| California | 8.85^***^ (6.88, 11.39) | 8.84^***^ (6.87, 11.37) | 31.90^***^ (27.41, 37.12) | 31.88^***^ (27.40, 37.10) |
| Colorado | 12.68^***^ (10.75, 14.97) | 12.67^***^ (10.74, 14.95) | 1.79^***^ (1.68, 1.90) | 1.79^***^ (1.68, 1.90) |
| Connecticut | NA | Excluded | 3.35^***^ (2.87, 3.92) | 3.35^***^ (2.87, 3.92) |
| Delaware | 0.92 (0.12, 6.85) | Excluded | 0.43^*^ (0.16, 1.17) | Excluded |
| District of Columbia | NA | Excluded | NA | Excluded |
| Georgia | 22.98^***^ (19.65, 26.87) | 22.96^***^ (19.63, 26.85) | 6.70^***^ (6.34, 7.09) | 6.70^***^ (6.34, 7.08) |
| Illinois | 0.15^***^ (0.05, 0.46) | 13.62^***^ (11.13, 16.68) | 5.88^***^ (5.29, 6.54) | 5.88^***^ (5.29, 6.54) |
| Indiana | 13.63^***^ (11.14, 16.69) | 7.41^***^ (5.29, 10.39) | 2.54^***^ (2.26, 2.86) | 2.54^***^ (2.26, 2.86) |
| Iowa | 7.41^***^ (5.29, 10.39) | 6.35^***^ (4.72, 8.55) | 2.49^***^ (2.09, 2.97) | 2.50^***^ (2.09, 2.97) |
| Kansas | 6.36^***^ (4.73, 8.55) | 2.02^***^ (1.68, 2.43) | 1.67^***^ (1.43, 1.94) | 1.67^***^ (1.43, 1.94) |
| Kentucky | 2.02^***^ (1.68, 2.43) | 22.96^***^ (19.63, 26.85) | 4.36^***^ (4.07, 4.67) | 4.36^***^ (4.07, 4.67) |
| Maine | NA | Excluded | NA | Excluded |
| Massachusetts | 1.40^***^ (1.12, 1.75) | 1.40^***^ (1.12, 1.75) | 1.83^***^ (1.70, 1.97) | 1.83^***^ (1.70, 1.97) |
| Michigan | 9.69^***^ (8.12, 11.56) | 9.69^***^ (8.11, 11.56) | 1.96^***^ (1.81, 2.12) | 1.96^***^ (1.81, 2.12) |
| Minnesota | 6.23^***^ (4.87, 7.98) | 6.23^***^ (4.87, 7.97) | 5.50^***^ (4.97, 6.09) | 5.50^***^ (4.97, 6.09) |
| Nevada | 1.18 (0.61, 2.28) | 1.17 (0.61, 2.28) | 11.59^***^ (9.87, 13.62) | 11.59^***^ (9.86, 13.61) |
| New Hampshire | NA | Excluded | 0.12^***^ (0.06, 0.23) | 0.12^***^ (0.06, 0.23) |
| New Jersey | 2.25^***^ (1.85, 2.74) | 2.25^***^ (1.85, 2.75) | 6.04^***^ (5.68, 6.42) | 6.04^***^ (5.68, 6.42) |
| New Mexico | 1.69^***^ (1.32, 2.18) | 1.69^***^ (1.32, 2.18) | 1.30^***^ (1.18, 1.42) | 1.30^***^ (1.18, 1.42) |
| New York | 5.66^***^ (4.58, 6.99) | 5.66^***^ (4.58, 6.99) | 1.17^***^ (1.06, 1.29) | 1.17^***^ (1.06, 1.29) |
| North Carolina | 0.16^***^ (0.10, 0.24) | 0.16^***^ (0.10, 0.24) | 2.36^***^ (2.22, 2.50) | 2.36^***^ (2.22, 2.50) |
| Ohio | 3.23^***^ (2.70, 3.86) | 3.23^***^ (2.70, 3.86) | 1.17^***^ (1.09, 1.26) | 1.17^***^ (1.09, 1.26) |
| Oklahoma | 0.26^***^ (0.18, 0.39) | 0.26^***^ (0.18, 0.39) | 0.09^***^ (0.07, 0.11) | 0.09^***^ (0.07, 0.11) |
| Oregon | 4.32^***^ (3.64, 5.12) | 4.32^***^ (3.64, 5.12) | 1.13^***^ (1.05, 1.22) | 1.13^***^ (1.05, 1.22) |
| Pennsylvania | 7.94^***^ (6.44, 9.78) | 7.93^***^ (6.44, 9.77) | 104.16^***^ (92.27, 117.60) | 104.15^***^ (92.25, 117.58) |
| Puerto Rico | 1.33 (0.38, 4.64) | Excluded | 1.15 (0.77, 1.72) | 1.15 (0.77, 1.72) |
| Rhode Island | NA | Excluded | 0.25^***^ (0.20, 0.31) | 0.25^***^ (0.20, 0.31) |
| South Carolina | 7.52^***^ (6.35, 8.91) | 7.52^***^ (6.35, 8.92) | 1.37^***^ (1.27, 1.47) | 1.37^***^ (1.27, 1.47) |
| Utah | 0.74^**^ (0.56, 0.99) | 0.74^**^ (0.55, 0.99) | 0.98 (0.90, 1.07) | 0.98 (0.90, 1.07) |
| Vermont | NA | Excluded | 0.07^***^ (0.02, 0.21) | Excluded |
| Virginia | 0.01^***^ (0.002, 0.04) | Excluded | 0.05^***^ (0.04, 0.05) | 0.05^***^ (0.04, 0.05) |
| Washington | 0.36^***^ (0.20, 0.68) | 0.36^***^ (0.20, 0.68) | 2.63^***^ (2.33, 2.97) | 2.63^***^ (2.33, 2.97) |
| West Virginia | 0.38 (0.09, 1.72) | Excluded | 0.23^***^ (0.12, 0.44) | 0.23^***^ (0.12, 0.44) |
| Wisconsin | 14.68^***^ (12.44, 17.33) | 14.68^***^ (12.44, 17.33) | 0.72^***^ (0.66, 0.79) | 0.72^***^ (0.66, 0.79) |
| **Incident Year** *(ref=2017)* |  |  |  |  |
| 2003 | 2.26^***^ (1.90, 2.68) | 2.23^***^ (1.87, 2.65) | 2.83^***^ (2.61, 3.07) | 2.83^***^ (2.61, 3.07) |
| 2004 | 0.59^***^ (0.51, 0.69) | 0.59^***^ (0.51, 0.68) | 2.42^***^ (2.26, 2.59) | 2.42^***^ (2.26, 2.59) |
| 2005 | 1.17^**^ (1.03, 1.33) | 1.17^**^ (1.02, 1.33) | 2.38^***^ (2.23, 2.55) | 2.38^***^ (2.23, 2.55) |
| 2006 | 1.36^***^ (1.21, 1.54) | 1.36^***^ (1.21, 1.54) | 1.40^***^ (1.31, 1.49) | 1.40^***^ (1.31, 1.49) |
| 2007 | 1.00 (0.88, 1.14) | 1.00 (0.88, 1.13) | 1.35^***^ (1.26, 1.44) | 1.35^***^ (1.26, 1.44) |
| 2008 | 0.95 (0.84, 1.07) | 0.94 (0.83, 1.07) | 0.80^***^ (0.75, 0.86) | 0.80^***^ (0.75, 0.86) |
| 2009 | 1.52^***^ (1.36, 1.70) | 1.52^***^ (1.36, 1.70) | 0.62^***^ (0.57, 0.66) | 0.62^***^ (0.57, 0.66) |
| 2010 | 1.00 (0.89, 1.12) | 1.00 (0.89, 1.12) | 0.90^***^ (0.84, 0.96) | 0.90^***^ (0.84, 0.96) |
| 2011 | 1.15^**^ (1.03, 1.28) | 1.14^**^ (1.03, 1.27) | 0.93^**^ (0.87, 1.00) | 0.93^**^ (0.87, 0.99) |
| 2012 | 0.81^***^ (0.72, 0.91) | 0.81^***^ (0.72, 0.90) | 0.73^***^ (0.68, 0.78) | 0.73^***^ (0.68, 0.78) |
| 2013 | 1.08 (0.96, 1.21) | 1.08 (0.96, 1.21) | 1.39^***^ (1.31, 1.48) | 1.39^***^ (1.31, 1.48) |
| 2014 | 0.82^***^ (0.73, 0.91) | 0.81^***^ (0.73, 0.90) | 1.37^***^ (1.30, 1.45) | 1.37^***^ (1.30, 1.45) |
| 2015 | 1.03 (0.94, 1.14) | 1.03 (0.94, 1.14) | 1.61^***^ (1.53, 1.70) | 1.61^***^ (1.53, 1.70) |
| 2016 | 0.97 (0.89, 1.06) | 0.97 (0.89, 1.06) | 1.08^***^ (1.03, 1.13) | 1.08^***^ (1.03, 1.13) |
|  | | | | |
| Observations | 233,108 | 209,332 | 233,108 | 231,782 |
| Log Likelihood | -32,487.68 | -32,412.47 | -90,079.39 | -90,043.51 |
| Akaike Inf. Crit. | 65,155.36 | 64,982.94 | 180,338.80 | 180,259.00 |
|  | | | | |
| Note:  ^*^p<0.1;^**^p<0.05;^***^p<0.01  ǂ In the analysis by missing status in CME narratives 11 states were excluded due to cell count <5: Connecticut, Delaware, District of Columbia, Illinois, Maine, New Hampshire, Puerto Rico, Rhode Island, Vermont, Virginia, West Virginia  ǂǂ In the analysis by missing status in LE narratives 4 states were excluded due to cell count <5: Delaware, District of Columbia, Maine, Vermont | | | | |
