# Supplementary material for: Not discussed: Inequalities in narrative text data for suicide deaths in the National Violent Death Reporting System
Source: PLoS One. 2021 Jul 16;16(7):e0254417. doi: 10.1371/journal.pone.0254417 (PMC8284808; doi:10.1371/journal.pone.0254417)
Supplement: S3 Table — (DOCX) [file pone.0254417.s004.docx]

| **S3 Table. Quasi-Poisson Regression of Character Length of NVDRS Narratives Predicted by Demographic Characteristics** | | | |  |
| --- | --- | --- | --- | --- |
|  | Incidence Rate Ratio (95% CI) | | |  |
|  |  | | |  |
|  | CME Narrative | LE Narrative | |  |
|  | | | |  |
| Constant | 787.61^***^ (767.45, 808.29) | 870.79^***^ (842.05, 900.50) | |  |
| **Incident Year** *(ref = 2017)* |  |  |  |  |
| 2003 | 0.44^***^ (0.43, 0.46) | 0.42^***^ (0.40, 0.44) | |  |
| 2004 | 0.50^***^ (0.49, 0.52) | 0.55^***^ (0.53, 0.57) | |  |
| 2005 | 0.53^***^ (0.52, 0.55) | 0.60^***^ (0.58, 0.63) | |  |
| 2006 | 0.54^***^ (0.52, 0.55) | 0.59^***^ (0.57, 0.61) | |  |
| 2007 | 0.57^***^ (0.55, 0.58) | 0.58^***^ (0.56, 0.60) | |  |
| 2008 | 0.61^***^ (0.60, 0.63) | 0.64^***^ (0.62, 0.67) | |  |
| 2009 | 0.65^***^ (0.63, 0.67) | 0.63^***^ (0.61, 0.65) | |  |
| 2010 | 0.77^***^ (0.75, 0.79) | 0.74^***^ (0.71, 0.76) | |  |
| 2011 | 0.81^***^ (0.79, 0.84) | 0.75^***^ (0.72, 0.76) | |  |
| 2012 | 0.81^***^ (0.79, 0.84) | 0.79^***^ (0.76, 0.82) | |  |
| 2013 | 0.86^***^ (0.83, 0.89) | 0.85^***^ (0.82, 0.89) | |  |
| 2014 | 0.89^***^ (0.85, 0.93) | 0.91^***^ (0.86, 0.96) | |  |
| 2015 | 0.91^***^ (0.87, 0.95) | 0.92^***^ (0.87, 0.96) | |  |
| 2016 | 0.93^***^ (0.89, 0.96) | 0.98 (0.94, 1.03) | |  |
| **Age** *(ref=40-49 years)* | |  |  |  |
| ≤18 | 1.06^***^ (1.06, 1.08) | 1.25^***^ (1.22, 1.28) | |  |
| 19-29 | 1.00 (0.99, 1.01) | 1.08^***^ (1.07, 1.10) | |  |
| 30-39 | 1.00 (0.99, 1.01) | 1.03^***^ (1.02, 1.04) | |  |
| 50-59 | 0.98^***^ (0.98, 0.99) | 0.95^***^ (0.94, 0.96) | |  |
| 60-69 | 0.97^***^ (0.96, 0.98) | 0.91^***^ (0.90, 0.92) | |  |
| 70-79 | 0.94^***^ (0.93, 0.95) | 0.87^***^ (0.86, 0.88) | |  |
| ≥80 | 0.93^***^ (0.91, 0.94) | 0.86^***^ (0.85, 0.88) | |  |
| Unknown/Missing | 0.84 (0.63, 1.11) | 0.60^**^ (0.39, 0.94) | |  |
| **Sex** *(ref=Male)* |  |  | |  |
| Female | 1.08^***^ (1.07, 1.08) | 1.05^***^ (1.04, 1.05) | |  |
| Unknown/Missing | 0.77^***^ (0.75, 0.78) | 1.52^***^ (1.48, 1.57) | |  |
| **Race or Ethnicity** *(ref=White)* | |  | |  |
| American Indian/Alaska Native | 1.05^***^ (1.01, 1.08) | 1.08^***^ (1.03, 1.12) | |  |
| Asian/Pacific Islander | 0.97^***^ (0.95, 0.99) | 0.93^***^ (0.90, 0.96) | |  |
| Black or African American | 0.94^***^ (0.93, 0.95) | 0.88^***^ (0.87, 0.90) | |  |
| Hispanic or Latino | 0.99 (0.98, 1.01) | 0.99 (0.97, 1.01) | |  |
| Other/Unspecified | 0.91^***^ (0.85, 0.98) | 0.93 (0.85, 1.02) | |  |
| Two or more races | 1.02 (1.00, 1.04) | 1.03 (0.99, 1.06) | |  |
| Unknown/Missing | 1.00 (0.87, 1.15) | 0.92 (0.79, 1.07) | |  |
| **Homelessness Status** *(ref=No)* | |  | |  |
| Yes | 1.08^***^ (1.05, 1.10) | 1.00 (0.96, 1.03) | |  |
| Unknown/Missing | 0.87^***^ (0.85, 0.89) | 0.88^***^ (0.86, 0.91) | |  |
| **Education Level** *(ref=High School or GED Diploma)* | |  | |  |
| 8th grade or less | 0.98^***^ (0.96, 0.99) | 0.95^***^ (0.93, 0.97) | |  |
| 9-12th grade, no diploma | 0.99^***^ (0.98, 1.00) | 0.96^***^ (0.95, 0.97) | |  |
| Some college, no degree | 1.02^***^ (1.01, 1.03) | 1.01 (0.99, 1.02) | |  |
| Associate's degree | 1.03^***^ (1.02, 1.04) | 1.03^***^ (1.01, 1.05) | |  |
| Bachelor's degree | 1.02^***^ (1.01, 1.03) | 1.02^**^ (1.00, 1.03) | |  |
| Master's degree | 1.03^***^ (1.02, 1.05) | 1.05^***^ (1.03, 1.08) | |  |
| Professional or Doctorate degree | 1.05^***^ (1.02, 1.07) | 1.05^***^ (1.02, 1.09) | |  |
| Unknown/Missing | 0.97^***^ (0.96, 0.98) | 1.03^***^ (1.02, 1.05) | |  |
| **Marital Status** *(ref=Married/In relationship)* | |  | |  |
| Divorced/Separated | 1.01 (1.00, 1.01) | 0.97^***^ (0.96, 0.98) | |  |
| Single/Never Married | 0.98^***^ (0.98, 0.99) | 0.94^***^ (0.93, 0.95) | |  |
| Widowed | 0.99 (0.98, 1.01) | 0.96^***^ (0.94, 0.97) | |  |
| Unknown/Missing | 0.97 (0.95, 1.00) | 0.92^***^ (0.88, 0.96) | |  |
| **Military Status** *(ref=No)* | |  | |  |
| Yes | 1.00 (1.00, 1.01) | 1.01^**^ (1.00, 1.02) | |  |
| Unknown/Missing | 0.90^***^ (0.88, 0.91) | 0.82^***^ (0.81, 0.84) | |  |
| **Autopsy Performed** *(ref=Yes)* | |  | |  |
| No | 0.96^***^ (0.95, 0.97) | 0.97^***^ (0.96, 0.98) | |  |
| Unknown/Missing | 0.91^***^ (0.86, 0.97) | 0.94^*^ (0.88, 1.00) | |  |
| **Place of Death** *(ref=Home)* | |  | |  |
| Hospice or LTC Facility | 1.01 (0.98, 1.04) | 0.86^***^ (0.81, 0.92) | |  |
| Hospital | 0.97^***^ (0.96, 0.98) | 0.98^***^ (0.97, 0.99) | |  |
| Other | 0.97^***^ (0.96, 0.97) | 0.97^***^ (0.96, 0.98) | |  |
| Unknown/Missing | 0.87^***^ (0.82, 0.91) | 0.91^***^ (0.86, 0.97) | |  |
|  | | | |  |
| Observations | 201,230 | 173,619 | |  |
|  | | | |  |
| Note:  ^*^p<0.1;^**^p<0.05;^***^p<0.01 | | | | |
