# Supplementary material for: Not discussed: Inequalities in narrative text data for suicide deaths in the National Violent Death Reporting System
Source: PLoS One. 2021 Jul 16;16(7):e0254417. doi: 10.1371/journal.pone.0254417 (PMC8284808; doi:10.1371/journal.pone.0254417)
Supplement: S4 Table — (DOCX) [file pone.0254417.s005.docx]

| **S4 Table. Quasi-Poisson Regression of Character Length of NVDRS Narratives Predicted by Demographic Characteristics, Excluding Outliers (Longest 1% of Narratives)** | | |  |
| --- | --- | --- | --- |
|  | | |  |
|  | Incidence Rate Ratio (95% CI) | |  |
|  |  | |  |
|  | CME Narrative | LE Narrative |  |
|  | | |  |
| **Constant** | 805.40^***^ (796.60, 814.30) | 766.76^***^ (756.14, 777.53) |  |
| **Age (years)** *(ref=”40-49”)* | |  |  |
| <= 18 | 1.03^***^ (1.02, 1.04) | 1.17^***^ (1.15, 1.19) |  |
| 19-29 | 0.99^*^ (0.99, 1.00) | 1.07^***^ (1.06, 1.08) |  |
| 30-39 | 1.00 (0.99, 1.01) | 1.03^***^ (1.02, 1.04) |  |
| 50-59 | 0.98^***^ (0.98, 0.99) | 0.95^***^ (0.95, 0.96) |  |
| 60-69 | 0.97^***^ (0.96, 0.98) | 0.93^***^ (0.92, 0.94) |  |
| 70-79 | 0.94^***^ (0.93, 0.95) | 0.89^***^ (0.88, 0.90) |  |
| >= 80 | 0.93^***^ (0.92, 0.95) | 0.88^***^ (0.87, 0.90) |  |
| Unknown/Missing | 1.00 (0.82, 1.22) | 1.03 (0.71, 1.49) |  |
| **Sex** *(ref=”Male”)* |  |  |  |
| Female | 1.07^***^ (1.06, 1.07) | 1.03^***^ (1.03, 1.04) |  |
| Unknown/Missing | 1.00 (0.49, 2.05) | 1.00 (0.40, 2.46) |  |
| **Race or Ethnicity** *(ref=”White”)* | |  |  |
| American Indian/Alaska Native | 0.97^***^ (0.95, 0.99) | 0.93^***^ (0.91, 0.95) |  |
| Asian/Pacific Islander | 0.99 (0.97, 1.00) | 0.93^***^ (0.91, 0.94) |  |
| Black or African American | 0.96^***^ (0.95, 0.97) | 0.91^***^ (0.90, 0.92) |  |
| Hispanic or Latino | 0.98^***^ (0.97, 0.99) | 0.94^***^ (0.93, 0.95) |  |
| Other/Unspecified | 0.97 (0.93, 1.02) | 0.94^**^ (0.89, 1.00) |  |
| Two or more races | 0.99 (0.98, 1.01) | 1.00 (0.98, 1.02) |  |
| Unknown/Missing | 0.98 (0.88, 1.10) | 0.93 (0.79, 1.08) |  |
| **Homelessness Status** *(ref=”No”)* | |  |  |
| Yes | 1.06^***^ (1.04, 1.08) | 0.96^***^ (0.94, 0.98) |  |
| Unknown/Missing | 0.91^***^ (0.90, 0.93) | 0.96^***^ (0.94, 0.98) |  |
| **Education Level** *(ref=”High School or GED Diploma”)* | |  |  |
| 8th grade or less | 0.98^**^ (0.97, 1.00) | 0.97^***^ (0.96, 0.99) |  |
| 9-12th grade, no diploma | 1.00 (0.99, 1.00) | 0.99^**^ (0.98, 1.00) |  |
| Some college, no degree | 1.01^***^ (1.01, 1.02) | 1.01^**^ (1.00, 1.02) |  |
| Associate's degree | 1.02^***^ (1.01, 1.03) | 1.01^*^ (1.00, 1.02) |  |
| Bachelor's degree | 1.01^**^ (1.00, 1.02) | 1.00 (0.99, 1.01) |  |
| Master's degree | 1.02^***^ (1.01, 1.04) | 1.02^***^ (1.01, 1.04) |  |
| Professional or Doctorate degree | 1.04^***^ (1.02, 1.06) | 1.05^***^ (1.03, 1.07) |  |
| 8th grade or less | 0.95^***^ (0.94, 0.96) | 1.03^***^ (1.02, 1.04) |  |
| **Marital Status** *(ref=”Married/In relationship”)* | |  |  |
| Divorced/Separated | 1.00 (1.00, 1.01) | 0.98^***^ (0.97, 0.98) |  |
| Single/Never Married | 0.99^***^ (0.98, 0.99) | 0.94^***^ (0.93, 0.94) |  |
| Widowed | 1.00 (0.99, 1.01) | 0.96^***^ (0.95, 0.97) |  |
| Unknown/Missing | 0.92^***^ (0.90, 0.94) | 0.87^***^ (0.85, 0.90) |  |
| **Military Status** *(ref=”No”)* | |  |  |
| Yes | 1.00 (0.99, 1.01) | 1.01^*^ (1.00, 1.01) |  |
| Unknown/Missing | 1.07^***^ (1.06, 1.08) | 1.03^***^ (1.01, 1.05) |  |
| **Autopsy Performed** *(ref=”Yes”)* | |  |  |
| No | 0.95^***^ (0.95, 0.96) | 0.99^***^ (0.98, 0.99) |  |
| Unknown/Missing | 0.98 (0.94, 1.02) | 1.08^***^ (1.03, 1.13) |  |
| **Place of Death** *(ref=”Home”)* | |  |  |
| Hospice or LTC Facility | 0.97^*^ (0.95, 1.00) | 0.86^***^ (0.83, 0.90) |  |
| Hospital | 0.99^***^ (0.98, 0.99) | 0.98^***^ (0.97, 0.99) |  |
| Other | 0.97^***^ (0.96, 0.97) | 0.96^***^ (0.95, 0.97) |  |
| Unknown/Missing | 0.95^***^ (0.91, 0.99) | 0.96 (0.92, 1.01) |  |
| **Reporting State** *(ref=”Maryland”)* | |  |  |
| Alaska | 1.67^***^ (1.65, 1.70) | 2.08^***^ (2.04, 2.12) |  |
| Arizona | 1.52^***^ (1.49, 1.54) | 2.30^***^ (2.26, 2.34) |  |
| California | 0.83^***^ (0.80, 0.85) | 0.59^***^ (0.55, 0.64) |  |
| Colorado | 1.09^***^ (1.08, 1.10) | 1.94^***^ (1.92, 1.96) |  |
| Connecticut | 0.55^***^ (0.53, 0.57) | 0.33^***^ (0.31, 0.35) |  |
| Delaware | 0.56^***^ (0.51, 0.62) | 1.53^***^ (1.42, 1.65) |  |
| District of Columbia | 1.60^***^ (1.46, 1.74) | 1.76^***^ (1.59, 1.95) |  |
| Georgia | 0.84^***^ (0.83, 0.85) | 0.75^***^ (0.74, 0.76) |  |
| Illinois | 1.45^***^ (1.42, 1.47) | 1.17^***^ (1.13, 1.20) |  |
| Indiana | 0.96^***^ (0.94, 0.98) | 1.05^***^ (1.03, 1.08) |  |
| Iowa | 0.87^***^ (0.85, 0.90) | 0.78^***^ (0.75, 0.81) |  |
| Kansas | 0.66^***^ (0.64, 0.67) | 0.91^***^ (0.88, 0.94) |  |
| Kentucky | 0.49^***^ (0.48, 0.50) | 0.24^***^ (0.23, 0.25) |  |
| Maine | 0.77^***^ (0.74, 0.80) | 1.04^*^ (1.00, 1.08) |  |
| Massachusetts | 0.70^***^ (0.69, 0.71) | 0.95^***^ (0.93, 0.96) |  |
| Michigan | 0.62^***^ (0.62, 0.63) | 0.96^***^ (0.94, 0.97) |  |
| Minnesota | 1.00 (0.98, 1.02) | 1.17^***^ (1.14, 1.20) |  |
| Nevada | 1.16^***^ (1.13, 1.20) | 0.89^***^ (0.84, 0.94) |  |
| New Hampshire | 1.46^***^ (1.42, 1.50) | 1.53^***^ (1.48, 1.58) |  |
| New Jersey | 1.06^***^ (1.05, 1.07) | 1.70^***^ (1.68, 1.73) |  |
| New Mexico | 1.25^***^ (1.23, 1.27) | 1.31^***^ (1.29, 1.34) |  |
| New York | 0.69^***^ (0.68, 0.70) | 1.06^***^ (1.04, 1.08) |  |
| North Carolina | 1.11^***^ (1.10, 1.12) | 1.18^***^ (1.16, 1.20) |  |
| Ohio | 0.87^***^ (0.86, 0.88) | 0.95^***^ (0.94, 0.97) |  |
| Oklahoma | 0.86^***^ (0.85, 0.87) | 0.86^***^ (0.84, 0.87) |  |
| Oregon | 1.26^***^ (1.25, 1.28) | 1.21^***^ (1.19, 1.23) |  |
| Pennsylvania | 0.78^***^ (0.76, 0.80) | 0.69^***^ (0.65, 0.74) |  |
| Puerto Rico | 0.30^***^ (0.27, 0.33) | 0.18^***^ (0.16, 0.21) |  |
| Rhode Island | 1.08^***^ (1.05, 1.10) | 1.15^***^ (1.12, 1.18) |  |
| South Carolina | 0.62^***^ (0.61, 0.63) | 0.72^***^ (0.71, 0.74) |  |
| Utah | 1.27^***^ (1.25, 1.28) | 1.44^***^ (1.42, 1.47) |  |
| Vermont | 1.00 (0.96, 1.04) | 0.65^***^ (0.61, 0.70) |  |
| Virginia | 0.78^***^ (0.77, 0.78) | 0.77^***^ (0.76, 0.78) |  |
| Washington | 1.57^***^ (1.54, 1.60) | 1.72^***^ (1.68, 1.76) |  |
| West Virginia | 1.02 (0.98, 1.06) | 1.23^***^ (1.18, 1.29) |  |
| Wisconsin | 1.16^***^ (1.15, 1.18) | 1.74^***^ (1.72, 1.76) |  |
| **Incident Year** *(ref=”2017”)* |  |  |  |
| 2003 | 0.46^***^ (0.45, 0.47) | 0.44^***^ (0.43, 0.45) |  |
| 2004 | 0.51^***^ (0.50, 0.52) | 0.50^***^ (0.49, 0.51) |  |
| 2005 | 0.53^***^ (0.52, 0.54) | 0.55^***^ (0.55, 0.56) |  |
| 2006 | 0.53^***^ (0.52, 0.54) | 0.55^***^ (0.54, 0.56) |  |
| 2007 | 0.56^***^ (0.55, 0.57) | 0.55^***^ (0.54, 0.56) |  |
| 2008 | 0.61^***^ (0.60, 0.62) | 0.62^***^ (0.61, 0.63) |  |
| 2009 | 0.64^***^ (0.63, 0.65) | 0.62^***^ (0.61, 0.63) |  |
| 2010 | 0.76^***^ (0.75, 0.77) | 0.71^***^ (0.71, 0.72) |  |
| 2011 | 0.80^***^ (0.80, 0.81) | 0.74^***^ (0.73, 0.75) |  |
| 2012 | 0.81^***^ (0.80, 0.82) | 0.78^***^ (0.77, 0.79) |  |
| 2013 | 0.85^***^ (0.84, 0.86) | 0.82^***^ (0.81, 0.83) |  |
| 2014 | 0.88^***^ (0.88, 0.89) | 0.87^***^ (0.86, 0.88) |  |
| 2015 | 0.89^***^ (0.89, 0.90) | 0.88^***^ (0.88, 0.89) |  |
| 2016 | 0.92^***^ (0.91, 0.92) | 0.96^***^ (0.95, 0.96) |  |
|  | | |  |
| Observations | 199,217 | 173,619 |  |
|  | | |  |
| Note:  ^*^p<0.1;^**^p<0.05;^***^p<0.01 | | | |
