# Supplementary material for: Not discussed: Inequalities in narrative text data for suicide deaths in the National Violent Death Reporting System
Source: PLoS One. 2021 Jul 16;16(7):e0254417. doi: 10.1371/journal.pone.0254417 (PMC8284808; doi:10.1371/journal.pone.0254417)
Supplement: S5 Table — (DOCX) [file pone.0254417.s006.docx]

| **S5 Table. Sensitivity Analyses for Logistic Regression of Missing Status for NVDRS Narratives Abstracted from Coroner/Medical Examiner (CME) Reports.** | | | | | |  |
| --- | --- | --- | --- | --- | --- | --- |
|  | OR (95% CI) | | | | |  |
|  | Model 1 | Model 2 | | Model 3 | Model 4 |  |
| Intercept | 0.03^***^ (0.03, 0.04) | 0.03^***^ (0.03, 0.03) | | 0.04^***^ (0.03, 0.05) | 0.05^***^ (0.04, 0.06) |  |
| **Incident Year** | 0.96^***^ (0.95, 0.97) | 0.97^***^ (0.96, 0.98) | | 0.96^***^ (0.95, 0.97) | 0.91^***^ (0.90, 0.92) |  |
| **Age (years, ref=”40-49”)** | |  | |  |  |  |
| <= 18 | 1.08 (0.93, 1.24) | 1.11 (0.96, 1.28) | | 0.99 (0.83, 1.17) | 1.05 (0.91, 1.21) |  |
| 19-29 | 1.03 (0.95, 1.12) | 1.03 (0.95, 1.12) | | 1.03 (0.94, 1.12) | 1.03 (0.95, 1.12) |  |
| 30-39 | 1.04 (0.97, 1.13) | 1.04 (0.97, 1.13) | | 1.04 (0.95, 1.13) | 1.04 (0.97, 1.12) |  |
| 50-59 | 1.05 (0.98, 1.13) | 1.05 (0.98, 1.13) | | 1.06 (0.98, 1.15) | 1.04 (0.97, 1.12) |  |
| 60-69 | 1.09^*^ (1.00, 1.19) | 1.09^*^ (1.00, 1.18) | | 1.07 (0.98, 1.17) | 1.05 (0.97, 1.15) |  |
| 70-79 | 1.26^***^ (1.14, 1.40) | 1.26^***^ (1.13, 1.40) | | 1.22^***^ (1.09, 1.37) | 1.16^***^ (1.04, 1.29) |  |
| >= 80 | 1.15^**^ (1.01, 1.31) | 1.15^**^ (1.01, 1.31) | | 1.14^*^ (0.99, 1.30) | 1.06^**^ (0.94, 1.20) |  |
| Unknown/Missing | 0.18^***^ (0.11, 0.31) | 0.19^***^ (0.11, 0.31) | | 0.14^***^ (0.07, 0.27) | 0.19^***^ (0.12, 0.31) |  |
| **Sex** *(ref=”Male”)* |  |  | |  |  |  |
| Female | 1.00 (0.95, 1.06) | 1.00 (0.95, 1.06) | | 0.99 (0.93, 1.05) | 1.01^*^(0.99, 1.11) |  |
| Unknown/Missing | 0.64 (0.31, 1.32) | 0.65 (0.32, 1.32) | | 0.88 (0.33, 2.32) | 0.62 (0.30, 1.27) |  |
| **Race or Ethnicity** *(ref=”White”)* | |  | |  |  |  |
| American Indian/Alaska Native | 2.09^***^ (1.68, 2.60) | 2.11^***^ (1.69, 2.62) | | 2.21^***^ (1.74, 2.81) | 1.92^***^ (1.53, 2.42) |  |
| Asian/Pacific Islander | 0.77^**^ (0.62, 0.96) | 0.76^**^ (0.61, 0.95) | | 0.72^***^ (0.56, 0.92) | 0.78^**^ (0.63, 0.97) |  |
| Black or African American | 0.85^***^ (0.77, 0.93) | 0.84^***^ (0.77, 0.92) | | 0.83^***^ (0.73, 0.93) | 0.81^***^ (0.74, 0.90) |  |
| Hispanic or Latino | 0.95 (0.84, 1.08) | 0.95 (0.84, 1.07) | | 0.97 (0.85, 1.10) | 0.95 (0.84, 1.08) |  |
| Other/Unspecified | 2.05^**^ (1.14, 3.69) | 2.02^**^ (1.13, 3.64) | | 1.98^**^ (1.07, 3.65) | 1.76^*^ (0.91, 3.40) |  |
| Two or more races | 0.72^**^ (0.57, 0.91) | 0.73^**^ (0.57, 0.92) | | 0.70^**^ (0.54, 0.92) | 0.74^**^ (0.59, 0.91) |  |
| Unknown/Missing | 3.04^***^ (1.79, 5.15) | 2.99^***^ (1.76, 5.06) | | 3.64^***^ (2.03, 6.51) | 3.59^***^ (2.02, 6.38) |  |
| **Homelessness Status** *(ref=”No”)* | |  | |  |  |  |
| Yes | 0.63^***^ (0.45, 0.87) | 0.64^***^ (0.46, 0.88) | | 0.64^**^ (0.42, 0.96) | 0.66^**^ (0.48, 0.92) |  |
| Unknown/Missing | 17.09^***^ (15.52, 18.82) | 17.17^***^ (15.59, 18.91) | | 15.71^***^ (14.11, 17.48) | 17.04^***^ (15.44, 18.80) |  |
| **Education Level** *(ref=”High School or GED Diploma”)* | |  | |  |  |  |
| 8th grade or less | 0.97 (0.86, 1.10) | 1.01 (0.91, 1.13) | | 0.92 (0.80, 1.07) | 0.95 (0.84, 1.08) |  |
| 9-12th grade, no diploma | 0.96 (0.88, 1.05) | 0.94 (0.87, 1.01) | | 0.95 (0.87, 1.04) | 0.96 (0.88, 1.05) |  |
| Some college, no degree | 0.84^***^ (0.77, 0.91) | 0.91^**^ (0.84, 0.99) | | 0.82^***^ (0.74, 0.90) | 0.84^***^ (0.77, 0.92) |  |
| Associate's degree | 0.97 (0.87, 1.09) | 1.00 (0.90, 1.11) | | 0.94 (0.84, 1.06) | 0.99 (0.88, 1.04) |  |
| Bachelor's degree | 0.75^***^ (0.68, 0.84) | 0.82^***^ (0.75, 0.91) | | 0.72^***^ (0.64, 0.81) | 0.77^***^ (0.69, 0.86) |  |
| Master's degree | 0.82^**^ (0.79, 0.98) | 0.85^**^ (0.74, 0.99) | | 0.79^***^ (0.67, 0.94) | 0.81^**^ (0.69, 0.96) |  |
| Professional or Doctorate degree | 0.66^**^ (0.51, 0.85) | 0.73^**^ (0.60, 0.88) | | 0.64^***^ (0.50, 0.83) | 0.64^**^ (0.50, 0.84) |  |
| Unknown/Missing | 0.79^***^ (0.73, 0.85) | -- | | 0.71^***^ (0.66, 0.78) | 0.76^***^ (0.70, 0.82) |  |
| **Marital Status** *(ref=”Married/In relationship”)* | |  | |  |  |  |
| Divorced/Separated | 0.91^**^ (0.85, 0.97) | 0.91^**^ (0.86, 0.97) | | 0.91^**^ (0.85, 0.97) | 0.91^**^ (0.86, 0.98) |  |
| Single/Never Married | 0.86^***^ (0.81, 0.92) | 0.86^***^ (0.81, 0.92) | | 0.86^***^ (0.80, 0.92) | 0.87^***^ (0.82, 0.93) |  |
| Widowed | 0.93 (0.84, 1.03) | 0.93 (0.84, 1.03) | | 0.93 (0.83, 1.04) | 0.95 (0.86, 1.06) |  |
| Unknown/Missing | 1.12 (0.85, 1.47) | 1.08 (0.84, 1.40) | | 1.38^*^ (0.96, 1.99) | 1.11 (0.83, 1.49) |  |
| **Military Status** *(ref=”No”)* | |  | |  |  |  |
| Yes | 1.10^**^ (1.03, 1.17) | 1.10^**^ (1.03, 1.17) | | 1.10^***^ (1.03, 1.18) | 1.09^**^ (1.02, 1.16) |  |
| Unknown/Missing | 1.22^***^ (1.11, 1.35) | 1.20^***^ (1.09, 1.32) | | 1.28^***^ (1.15, 1.42) | 1.18^***^ (1.07, 1.30) |  |
| **Autopsy Performed** *(ref=”Yes”)* | |  | |  |  |  |
| No | 1.85^***^ (1.72, 2.00) | 1.85^***^ (1.72, 2.00) | | 1.83^***^ (1.67, 1.99) | 1.55^***^ (1.38, 1.73) |  |
| Unknown/Missing | 3.30^***^ (2.25, 4.83) | 3.34^***^ (2.29, 4.87) | | 3.06^***^ (2.02, 4.64) | 2.63^***^ (1.72, 4.03) |  |
| **Place of Death (ref=”Home”)** | |  | |  |  |  |
| Hospice or LTC Facility | 1.43^***^ (1.21, 1.69) | 1.41^***^ (1.19, 1.66) | | 1.51^***^ (1.26, 1.80) | 1.34^***^ (1.13, 1.58) |  |
| Hospital | 1.05 (0.98, 1.13) | 1.05 (0.98, 1.13) | | 1.02 (0.94, 1.11) | 1.04 (0.96, 1.13) |  |
| Other | 0.92^**^ (0.86, 0.99) | 0.92^**^ (0.86, 0.98) | | 0.95 (0.88, 1.01) | 0.92^**^ (0.86, 0.99) |  |
| Unknown/Missing | 4.84^***^ (3.12, 7.49) | 4.81^***^ (3.11, 7.45) | | 5.64^***^ (3.42, 9.31) | 4.90^***^ (3.47, 6.93) |  |
| **Toxicology Report** *(ref=”No/NA”)* | -- | -- | | -- | 3.78^***^ (2.92, 4.89) |  |
|  |  | |  | | |  |
| Observations | 233,108 | 233,108 | | 195,343 | 233,108 |  |
|  |  | |  | | |  |

Note: ^*^p<0.1;^**^p<0.05;^***^p<0.01

Model 1: main analysis. Model 2: missing data imputed by multivariate chain equations in education status. Model 3: data restricted to single suicides only (no undetermined deaths, n=30,094). Model 4: additional adjustment by toxicology report.
