# Supplementary material for: Not discussed: Inequalities in narrative text data for suicide deaths in the National Violent Death Reporting System
Source: PLoS One. 2021 Jul 16;16(7):e0254417. doi: 10.1371/journal.pone.0254417 (PMC8284808; doi:10.1371/journal.pone.0254417)
Supplement: S6 Table — (DOCX) [file pone.0254417.s007.docx]

| **S6 Table. Sensitivity Analyses for Logistic Regression of Missing Status for NVDRS Narratives Abstracted from Law Enforcement (LE) Reports.** | | | | | |  |
| --- | --- | --- | --- | --- | --- | --- |
|  | OR (95% CI) | | | | |  |
|  | Model 1 | Model 2 | | Model 3 | Model 4 |  |
| Intercept | 0.25^***^ (0.23, 0.27) | 0.28^***^ (0.27, 0.30) | | 0.23^***^ (0.22, 0.25) | 0.25^***^ (0.24, 0.27) |  |
| **Incident Year** | 0.91^***^ (0.91, 0.92) | 0.91^***^ (0.90, 0.91) | | 0.92^***^ (0.91, 0.92) | 0.91^***^ (0.91, 0.92) |  |
| **Age (years, ref=”40-49”)** | |  | |  |  |  |
| <= 18 | 0.84^***^ (0.77, 0.91) | 0.81^***^ (0.75, 0.88) | | 0.82^***^ (0.74, 0.91) | 0.84^***^ (0.77, 0.91) |  |
| 19-29 | 0.89^***^ (0.85, 0.94) | 0.89^***^ (0.85, 0.93) | | 0.95 (0.91, 1.01) | 0.89^***^ (0.86, 0.94) |  |
| 30-39 | 0.95^**^ (0.91, 0.99) | 0.95^**^ (0.91, 0.99) | | 0.98 (0.94, 1.03) | 0.95^**^ (0.91, 0.99) |  |
| 50-59 | 1.00 (0.96, 1.04) | 1.00 (0.96, 1.04) | | 1.01 (0.96, 1.06) | 1.00 (0.96, 1.04) |  |
| 60-69 | 1.04 (0.99, 1.09) | 1.04 (0.99, 1.09) | | 1.04 (0.98, 1.10) | 1.03 (0.99, 1.09) |  |
| 70-79 | 1.07^**^ (1.01, 1.14) | 1.07^**^ (1.01, 1.14) | | 1.10^***^ (1.03, 1.18) | 1.07^**^ (1.00, 1.13) |  |
| >= 80 | 1.03 (0.96, 1.12) | 1.03 (0.96, 1.11) | | 1.06 (0.97, 1.15) | 1.03 (0.95, 1.11) |  |
| Unknown/Missing | 0.43^***^ (0.31, 0.59) | 0.43^***^ (0.31, 0.59) | | 0.27^***^ (0.17, 0.45) | 0.43^***^ (0.31, 0.60) |  |
| **Sex** *(ref=”Male”)* |  |  | |  |  |  |
| Female | 1.12^***^ (1.09, 1.16) | 1.12^***^ (1.09, 1.16) | | 1.10^***^ (1.06, 1.14) | 1.12^***^ (1.09, 1.16) |  |
| Unknown/Missing | 0.76 (0.46, 1.27) | 0.74 (0.44, 1.24) | | 1.03 (0.50, 2.12) | 0.77 (0.46, 1.27) |  |
| **Race or Ethnicity** *(ref=”White”)* | |  | |  |  |  |
| American Indian/Alaska Native | 1.76^***^ (1.49, 2.08) | 1.75^***^ (1.48, 2.07) | | 1.95^***^ (1.63, 2.34) | 1.75^***^ (1.48, 2.06) |  |
| Asian/Pacific Islander | 1.13^*^ (1.00, 1.28) | 1.13^*^ (1.00, 1.28) | | 1.13^*^ (1.00, 1.29) | 1.13^*^ (1.00, 1.28) |  |
| Black or African American | 1.17^***^ (1.11, 1.23) | 1.17^***^ (1.11, 1.23) | | 1.06 (0.98, 1.13) | 1.17^***^ (1.11, 1.23) |  |
| Hispanic or Latino | 1.11^**^ (1.04, 1.19) | 1.11^**^ (1.03, 1.19) | | 1.12^**^ (1.03, 1.21) | 1.11^***^ (1.04, 1.19) |  |
| Other/Unspecified | 1.59^**^ (1.12, 2.26) | 1.61^**^ (1.14, 2.27) | | 1.63^**^ (1.18, 2.24) | 1.58^**^ (1.11, 2.25) |  |
| Two or more races | 0.79^***^ (0.71, 0.88) | 0.77^***^ (0.70, 0.86) | | 0.81^***^ (0.71, 0.93) | 0.79^***^ (0.71, 0.88) |  |
| Unknown/Missing | 3.24^***^ (2.28, 4.61) | 3.22^***^ (2.27, 4.57) | | 3.77^***^ (2.55, 5.59) | 3.27^***^ (2.30, 4.66) |  |
| **Homelessness Status** *(ref=”No”)* | |  | |  |  |  |
| Yes | 0.80^***^ (0.70, 0.91) | 0.80^***^ (0.70, 0.91) | | 0.79^**^ (0.65, 0.95) | 0.80^***^ (0.70, 0.91) |  |
| Unknown/Missing | 4.12^***^ (3.82, 4.45) | 4.11^***^ (3.81, 4.43) | | 4.18^***^ (3.85, 4.55) | 4.11^***^ (3.82, 4.44) |  |
| **Education Level** *(ref=”High School or GED Diploma”)* | |  | |  |  |  |
| 8th grade or less | 1.12^**^ (1.04, 1.20) | 1.13^**^ (1.06, 1.20) | | 1.02 (0.93, 1.13) | 1.11^***^ (1.04, 1.20) |  |
| 9-12th grade, no diploma | 0.94^**^ (0.89, 0.99) | 0.97 (0.93, 1.01) | | 0.95^*^ (0.89, 1.01) | 0.94^**^ (0.89, 0.99) |  |
| Some college, no degree | 0.94^**^ (0.89, 0.99) | 0.98 (0.94, 1.03) | | 0.94^*^ (0.88, 1.00) | 0.94^**^ (0.89, 0.99) |  |
| Associate's degree | 0.95 (0.89, 1.03) | 0.99 (0.93, 1.05) | | 0.96 (0.89, 1.05) | 0.96 (0.89, 1.03) |  |
| Bachelor's degree | 0.92^**^ (0.86, 0.99) | 0.94^**^ (0.89, 1.00) | | 0.92^**^ (0.85, 0.99) | 0.92^**^ (0.86, 0.99) |  |
| Master's degree | 0.96 (0.86, 1.06) | 0.96 (0.89, 1.04) | | 0.96 (0.86, 1.08) | 0.96 (0.86, 1.06) |  |
| Professional or Doctorate degree | 0.88^*^ (0.76, 1.02) | 0.92 (0.83, 1.02) | | 0.86^*^ (0.73, 1.02) | 0.88 (0.76, 1.02) |  |
| Unknown/Missing | 1.19^***^ (1.14, 1.24) | -- | | 1.25^***^ (1.19, 1.31) | 1.19^***^ (1.14, 1.24) |  |
| **Marital Status** *(ref=”Married/In relationship”)* | |  | |  |  |  |
| Divorced/Separated | 0.99 (0.96, 1.03) | 0.99 (0.96, 1.03) | | 0.99 (0.95, 1.03) | 1.00 (0.96, 1.03) |  |
| Single/Never Married | 0.99 (0.95, 1.03) | 0.99 (0.95, 1.03) | | 0.98 (0.93, 1.01) | 0.99 (0.95, 1.03) |  |
| Widowed | 1.05 (0.99, 1.12) | 1.05 (0.99, 1.12) | | 1.04 (0.97, 1.12) | 1.05 (0.99, 1.12) |  |
| Unknown/Missing | 0.91 (0.80, 1.04) | 0.97 (0.85, 1.11) | | 0.91 (0.76, 1.08) | 0.91 (0.80, 1.04) |  |
| **Military Status** *(ref=”No”)* | |  | |  |  |  |
| Yes | 0.98 (0.94, 1.02) | 0.98 (0.94, 1.02) | | 0.97 (0.93, 1.01) | 0.97 (0.94, 1.02) |  |
| Unknown/Missing | 1.29^***^ (1.22, 1.36) | 1.33^***^ (1.26, 1.40) | | 1.17^***^ (1.09, 1.24) | 1.29^***^ (1.22, 1.36) |  |
| **Autopsy Performed** *(ref=”Yes”)* | |  | |  |  |  |
| No | 1.06^**^ (1.02, 1.10) | 1.06^**^ (1.02, 1.10) | | 1.04^*^ (1.00, 1.09) | 1.04^**^ (1.00 1.09) |  |
| Unknown/Missing | 1.29^*^ (0.97, 1.70) | 1.28^*^ (0.97, 1.69) | | 1.31 (0.94, 1.82) | 1.26 (0.95, 1.67) |  |
| **Place of Death** *(ref=”Home”)* | |  | |  |  |  |
| Hospice or LTC Facility | 3.59^***^ (3.21, 4.01) | 3.61^***^ (3.23, 4.04) | | 3.28^***^ (2.90, 3.71) | 3.59^***^ (3.21, 4.02) |  |
| Hospital | 1.94^***^ (1.85, 2.03) | 1.93^***^ (1.84, 2.0\2) | | 1.69^***^ (1.60, 1.78) | 1.93^***^ (1.85, 2.02) |  |
| Other | 1.16^***^ (1.12, 1.20) | 1.16^***^ (1.12, 1.20) | | 1.16^***^ (1.11, 1.21) | 1.16^***^ (1.12, 1.20) |  |
| Unknown/Missing | 2.73^***^ (1.98, 3.76) | 2.73^***^ (1.98, 3.75) | | 2.93^***^ (2.07, 4.15) | 2.71^***^ (1.99, 3.71) |  |
| **Toxicology Report** *(ref=”No/NA”)* | -- | -- | | -- | 1.12^**^ (1.02, 1.24) |  |
| Observations | 233,108 | 233,108 | | 195,343 | 233,108 |  |
|  |  | |  | | |  |

Note:^*^p<0.1;^**^p<0.05;^***^p<0.01

Model 1: main analysis. Model 2: missing data imputed by multivariate chain equations in education status. Model 3: data restricted to single suicides only (no undetermined deaths, n=30,094). Model 4: additional adjustment by toxicology report.
