# Supplementary material for: Not discussed: Inequalities in narrative text data for suicide deaths in the National Violent Death Reporting System
Source: PLoS One. 2021 Jul 16;16(7):e0254417. doi: 10.1371/journal.pone.0254417 (PMC8284808; doi:10.1371/journal.pone.0254417)
Supplement: S7 Table — (DOCX) [file pone.0254417.s008.docx]

| **S7 Table. Sensitivity Analyses of Quasi-Poisson Regression of Character Length of NVDRS Narratives Abstracted from Coroner/Medical Examiner (CME) Reports.** | | | | | |  |
| --- | --- | --- | --- | --- | --- | --- |
|  | Mean Count Ratio (95% CI) | | | | |  |
|  | Model 1 | Model 2 | | Model 3 | Model 4 |  |
| Intercept | 351.32^***^ (346.50,356.22) | 343.04^***^ (338.93,347.20) | | 353.22^***^ (347.83,358.68) | 345.80^***^ (340.94,350.74) |  |
| **Incident Year** | 1.06^***^ (1.06, 1.06) | 1.06^***^ (1.06, 1.06) | | 1.06^***^ (1.06, 1.07) | 1.06^***^ (1.06, 1.07) |  |
| **Age (years, ref=”40-49”)** | |  | |  |  |  |
| <= 18 | 1.06^***^ (1.06, 1.06) | 1.07^***^ (1.05, 1.09) | | 1.05^***^ (1.03, 1.07) | 1.06^***^ (1.05, 1.08) |  |
| 19-29 | 1.00 (1.05, 1.08) | 1.00 (0.99, 1.01) | | 1.00 (0.99, 1.01) | 1.00 (0.99, 1.01) |  |
| 30-39 | 1.00 (0.99, 1.01) | 1.00 (0.99, 1.01) | | 1.00 (0.99, 1.01) | 1.00 (0.99, 1.01) |  |
| 50-59 | 0.98^***^ (0.98, 0.99) | 0.98^***^ (0.98, 0.99) | | 0.98^***^ (0.98, 0.99) | 0.98^***^ (0.98, 0.99) |  |
| 60-69 | 0.97^***^ (0.96, 0.98) | 0.97^***^ (0.96, 0.98) | | 0.96^***^ (0.96, 0.97) | 0.97^***^ (0.96, 0.98) |  |
| 70-79 | 0.94^***^ (0.93, 0.95) | 0.94^***^ (0.93, 0.95) | | 0.94^***^ (0.92, 0.95) | 0.95^***^ (0.94, 0.96) |  |
| >= 80 | 0.93^***^ (0.92, 0.94) | 0.93^***^ (0.92, 0.94) | | 0.92^***^ (0.91, 0.94) | 0.93^***^ (0.92, 0.95) |  |
| Unknown/Missing | 0.85 (0.65, 1.13) | 0.85 (0.65, 1.12) | | 0.69 (0.45, 1.04) | 0.85 (0.64, 1.12) |  |
| **Sex** *(ref=”Male”)* |  |  | |  |  |  |
| Female | 1.08^***^ (1.07, 1.08) | 1.08^***^ (1.07, 1.08) | | 1.08^***^ (1.07, 1.08) | 1.07^***^ (1.07, 1.08) |  |
| Unknown/Missing | 0.78^***^ (0.76, 0.79) | 0.78^***^ (0.76, 0.79) | | 0.69^***^ (0.68, 0.71) | 0.78^***^ (0.76, 0.79) |  |
| **Race or Ethnicity** *(ref=”White”)* | |  | |  |  |  |
| American Indian/Alaska Native | 1.05^***^ (1.02, 1.09) | 1.05^***^ (1.02, 1.09) | | 1.04^**^ (1.00, 1.07) | 1.06^***^ (1.02, 1.09) |  |
| Asian/Pacific Islander | 0.97^***^ (0.95, 0.99) | 0.97^***^ (0.95, 0.99) | | 0.97^***^ (0.95, 0.99) | 0.97^***^ (0.95, 0.99) |  |
| Black or African American | 0.94^***^ (0.93, 0.95) | 0.94^***^ (0.93, 0.95) | | 0.95^***^ (0.94, 0.97) | 0.94^***^ (0.93, 0.95) |  |
| Hispanic or Latino | 0.99 (0.98, 1.01) | 1.00 (0.98, 1.01) | | 1.00 (0.98, 1.01) | 0.99 (0.98, 1.01) |  |
| Other/Unspecified | 0.92^***^ (0.86, 0.98) | 0.91^***^ (0.85, 0.97) | | 0.93^**^ (0.86, 0.99) | 0.92^***^ (0.86, 0.98) |  |
| Two or more races | 1.02 (1.00, 1.04) | 1.02 (1.00, 1.05) | | 1.03 (1.00, 1.05) | 1.02 (1.00, 1.04) |  |
| Unknown/Missing | 1.00 (0.87, 1.15) | 1.00 (0.87, 1.15) | | 0.96 (0.83, 1.11) | 1.00 (0.87, 1.15) |  |
| **Homelessness Status** *(ref=”No”)* | |  | |  |  |  |
| Yes | 1.08^***^ (1.05, 1.10) | 1.08^***^ (1.05, 1.10) | | 1.09^***^ (1.06, 1.13) | 1.07^***^ (1.05, 1.10) |  |
| Unknown/Missing | 0.87^***^ (0.85, 0.89) | 0.87^***^ (0.85, 0.89) | | 0.86^***^ (0.84, 0.88) | 0.87^***^ (0.85, 0.89) |  |
| **Education Level** *(ref=”High School or GED Diploma”)* | |  | |  |  |  |
| 8th grade or less | 0.98^***^ (0.96, 0.99) | 0.99 (0.98, 1.00) | | 0.97^***^ (0.95, 0.98) | 0.98^***^ (0.96, 0.99) |  |
| 9-12th grade, no diploma | 0.99^***^ (0.98, 1.00) | 0.99^***^ (0.98, 1.00) | | 0.99^**^ (0.98, 1.00) | 0.99^***^ (0.98, 1.00) |  |
| Some college, no degree | 1.02^***^ (1.01, 1.03) | 1.02^***^ (1.01, 1.03) | | 1.01^***^ (1.01, 1.02) | 1.02^***^ (1.01, 1.02) |  |
| Associate's degree | 1.03^***^ (1.02, 1.04) | 1.04^***^ (1.03, 1.06) | | 1.02^***^ (1.01, 1.04) | 1.03^***^ (1.02, 1.04) |  |
| Bachelor's degree | 1.02^***^ (1.01, 1.03) | 1.03^***^ (1.02, 1.04) | | 1.02^***^ (1.01, 1.03) | 1.02^***^ (1.01, 1.03) |  |
| Master's degree | 1.03^***^ (1.02, 1.05) | 1.05^***^ (1.03, 1.06) | | 1.03^***^ (1.02, 1.05) | 1.03^***^ (1.01, 1.04) |  |
| Professional or Doctorate degree | 1.05^***^ (1.02, 1.07) | 1.03^***^ (1.02, 1.05) | | 1.05^***^ (1.02, 1.07) | 1.04^***^ (1.02, 1.07) |  |
| Unknown/Missing | 0.97^***^ (0.96, 0.98) | -- | | 0.97^***^ (0.96, 0.99) | 0.97^***^ (0.96, 0.98) |  |
| **Marital Status** *(ref=”Married/In relationship”)* | |  | |  |  |  |
| Divorced/Separated | 1.01 (1.00, 1.01) | 1.01 (1.00, 1.01) | | 1.01 (1.00, 1.01) | 1.01 (1.00, 1.01) |  |
| Single/Never Married | 0.98^***^ (0.98, 0.99) | 0.99^***^ (0.98, 0.99) | | 0.99^**^ (0.98, 1.00) | 0.98^***^ (0.98, 0.99) |  |
| Widowed | 0.99 (0.98, 1.01) | 1.00 (0.99, 1.01) | | 0.99 (0.98, 1.01) | 0.99 (0.98, 1.01) |  |
| Unknown/Missing | 0.97 (0.95, 1.00) | 0.97^**^ (0.94, 0.99) | | 0.98 (0.94, 1.01) | 0.97 (0.95, 1.00) |  |
| **Military Status** *(ref=”No”)* | |  | |  |  |  |
| Yes | 1.00 (1.00, 1.01) | 1.00 (0.99, 1.01) | | 1.00 (1.00, 1.01) | 1.00 (1.00, 1.01) |  |
| Unknown/Missing | 0.89^***^ (0.88, 0.91) | 0.89^***^ (0.88, 0.90) | | 0.88^***^ (0.87, 0.90) | 0.89^***^ (0.88, 0.91) |  |
| **Autopsy Performed** *(ref=”Yes”)* | |  | |  |  |  |
| No | 0.97^***^ (0.96, 0.97) | 0.97^***^ (0.96, 0.97) | | 0.97^***^ (0.96, 0.97) | 0.98^***^ (0.97, 0.99) |  |
| Unknown/Missing | 0.91^***^ (0.86, 0.97) | 0.91^***^ (0.86, 0.97) | | 0.90^***^ (0.85, 0.95) | 0.93^**^ (0.88, 0.99) |  |
| **Place of Death (ref=”Home”)** | |  | |  |  |  |
| Hospice or LTC Facility | 1.01 (0.98, 1.04) | 1.01 (0.98, 1.04) | | 0.97 (0.94, 1.01) | 1.02 (0.99, 1.05) |  |
| Hospital | 0.97^***^ (0.96, 0.98) | 0.97^***^ (0.96, 0.98) | | 0.97^***^ (0.96, 0.98) | 0.97^***^ (0.96, 0.98) |  |
| Other | 0.97^***^ (0.96, 0.97) | 0.97^***^ (0.96, 0.98) | | 0.97^***^ (0.96, 0.98) | 0.97^***^ (0.96, 0.97) |  |
| Unknown/Missing | 0.87^***^ (0.82, 0.91) | 0.87^***^ (0.82, 0.91) | | 0.85^***^ (0.80, 0.89) | 0.86^***^ (0.82, 0.91) |  |
| **Toxicology Report** *(ref=”No/NA”)* | -- | -- | | -- | 0.90^***^ (0.89, 0.92) |  |
|  |  | |  | | |  |
| Observations | 201,230 | 201,230 | | 171,442 | 201,230 |  |
|  |  | |  | | |  |

Note:^*^p<0.1;^**^p<0.05;^***^p<0.01

Model 1: main analysis. Model 2: missing data imputed by multivariate chain equations in education status. Model 3: data restricted to single suicides only (no undetermined deaths, n=30,094). Model 4: additional adjustment by toxicology report
