# Supplementary material for: Not discussed: Inequalities in narrative text data for suicide deaths in the National Violent Death Reporting System
Source: PLoS One. 2021 Jul 16;16(7):e0254417. doi: 10.1371/journal.pone.0254417 (PMC8284808; doi:10.1371/journal.pone.0254417)
Supplement: Attachment — Submitted filename: PLOS One Response Letter.docx [file pone.0254417.s010.docx]

**Comments from the editor**

1. **Two additional critiques I would highlight would be the issue of the relationship of LE/CME missingness to missingness of other data (Reviewer 1) and the relationship of these findings to the larger issue of missing data in other official records (Reviewer 2). Finally, a comment of my own -- it would be helpful to know the extent of overlap of the two types of missing (and nonmissing) data.**

Thank you for this comment. We woud like to clarify that our dataset includes three “types” of missing data: (1) Observations with a narrative character length of zero (C/ME, LE, or both) or whose circumstances were coded as “not known” by the NVDRS abtractors; (2) Observations whose narrative status was assigned as missing by the investigators based on having <31 characters, even though circumstances were coded as ‘known’ by the NVDRS abstractors; and (3) missing data on the predictors (age, sex, education, etc.).

To illustrate the extent of overlap in missingness for the first two types, have added the variable “Circumstances known” to the bottom of **Table 1**. It illustrates that of those observations whose C/ME narrative is coded as “missing,” 62.2% are missing because the circumstances were not known (first type of missing data) and 37.8% were coded as missing by the investigators because the narratives were <31 characters long (second type of missing data). These proportions are roughly reversed for the LE narratives. Overall, 6,170 (3%) observations were missing **both** C/ME and LE narratives (which we now state in the text - see Methods, Analysis 1).

This third type of missing data (missing data on predictors) is addressed in our response to Reviewer #1, comment #2.

The issue of missing data in mortality/administrative records more generally is addressed in our response to Reviewer #2, comments #6 and 7.

2. **Please ensure that your manuscript meets PLOS ONE's style requirements, including those for file naming. The PLOS ONE style templates can be found at https://journals.plos.org/plosone/s/file?id=wjVg/PLOSOne_formatting_sample_main_body.pdf and** [**https://journals.plos.org/plosone/s/file?id=ba62/PLOSOne_formatting_sample_title_authors_affiliations.pdf**](https://journals.plos.org/plosone/s/file?id=ba62/PLOSOne_formatting_sample_title_authors_affiliations.pdf)

We have made these style changes, as requested.

3. **We note that you have indicated that data from this study are available upon request. PLOS only allows data to be available upon request if there are legal or ethical restrictions on sharing data publicly. For information on unacceptable data access restrictions, please see** [**http://journals.plos.org/plosone/s/data-availability#loc-unacceptable-data-access-restrictions**](http://journals.plos.org/plosone/s/data-availability#loc-unacceptable-data-access-restrictions)**.**

**In your revised cover letter, please address the following prompts: a) If there are ethical or legal restrictions on sharing a de-identified data set, please explain them in detail (e.g., data contain potentially identifying or sensitive patient information) and who has imposed them (e.g., an ethics committee). Please also provide contact information for a data access committee, ethics committee, or other institutional body to which data requests may be sent. Or b) If there are no restrictions, please upload the minimal anonymized data set necessary to replicate your study findings as either Supporting Information files or to a stable, public repository and provide us with the relevant URLs, DOIs, or accession numbers. Please see http://www.bmj.com/content/340/bmj.c181.long for guidelines on how to de-identify and prepare clinical data for publication. For a list of acceptable repositories, please see** [**http://journals.plos.org/plosone/s/data-availability#loc-recommended-repositories**](http://journals.plos.org/plosone/s/data-availability#loc-recommended-repositories)**.**

We have responded to these prompts in the revised cover letter, as requested.

**Reviewer #1 Comments to the Author**

This paper is well-written, engaging to read, and addresses a basic (but important) question about the quality of NVDRS narrative data. With a few methodological changes, discussed below, I think that this paper contributes to the existing literature and may help current NVDRS abstractors address inequalities in data collection. Before I continue, I want to preface this review by clarifying that my current knowledge of the NVDRS system is on the abstraction side--I work with a state NVDRS and SUDORS (an overdose-specific subset of the NVDRS) team to improve their data quality. This means that I have a good knowledge of the data itself, but less knowledge about the data version your team for analysis.

1. **The first central methodological issue I see in this paper is a failure to fully address the state level variations in the data. Simply including a state dummy variable is not sufficient, as it does not address the fact that error terms will be clustered at the state level. While you acknowledge that there is state-level variation in the data, you need to build in either clustered error effects or potentially use a hierarchical model to fully address the variation. The NVDRS entry system is complex, and states have developed a wide variety of methodologies for submitting their data. Further, the difficulties of centralized versus local authority reporting means that some states may get electronic data downloads with CME reports (which often include written narratives), while others rely on scanned PDFs and fully manual abstraction. Finally, these reports are on tight schedules, so states that rely on fully manual abstraction may have less time to develop lengthy narratives. In short, I suspect state-level factors may be even more important than you suggested, and need a more robust inclusion in the model. If I were you, I would run a hierarchical model clustering at the state level, with the same outcomes/distributions.**

Thank you for this comment. You are correct that there is significant clustering by state, which we quantified using the intraclass correlation coefficient (ICC), which quantifies the variation between vs. within states.

ICC for C/ME missingness: 0.57, for LE missingness: 0.48

As a comparison, the ICC for clustering of C/ME missingness by incident year was only 0.01 and for LE missingness was only 0.03. This illustrates that the amount of missingness in narratives clusters within states, but not within years (time). As a result, incident year was included as a covariate in all models of narrative missingness, and state was used as a clustering variable.

ICC for C/ME length: 0.35, for LE length: 0.43

As a comparison, the ICC for clustering of C/ME length by incident year was only 0.18 and for LE missingness was only 0.11. This illustrates that the length of narratives clusters within states, with some modest clustering within years (time). As a result, incident year was included as a covariate in all models of narrative length, and state was used as a clustering variable.

In response, we we considered the following methods: 1) Quasipoisson model with robust standard errors, 2) multilevel modeling (in our case, Generalized Linear Mixed Model (GLMM)) and bootstrap while having state and year as random effects, and 3) a generalized estimating equation (GEE) with state as clusters, assuming exchangeable correlation structure and using sandwich estimator to be robustness of misspecification. All of the options are appropriate to address the violation of independence and homogeneity of variance. The first option works the best when the source of heteroscedasticity is unknown. However, in our case we know that the data came in clusters (state).

Both the second option and third option are appropriate for our research question and data. GEE and GLMM in general give very similar results. The difference of the two is that GEE gives the population (here, state) averaged estimates of parameters and GLMM gives individual estimates (here, state specific) averaged estimates of parameters. Since we are not interested in estimating the state specific estimates of parameters, using GEE tends to be a common choice. GEE in general requires a large number of clusters ~40, and the NVDRS dataset has 37 states as clusters.

Therefore, we refit our regression models using GEE logistic (for narrative missingness) and quasi-poisson (for narrative length, conditional on non-missingness) models with state as clusters, assuming exchangeable correlation structure and using sandwich estimator to be robustness of misspecification.

We have updated the Methods and the text, tables and figures of the Results using these GEE models. We note that the point estimates are substantially unchanged from the first manuscript, but now the standard errors reflect the clustering of observations within states. Please see the revised manuscript.

Also please see our response to Reviewer #2, comment #6 in which we discuss the importance of local, state, and federal stakeholders to collaborate to identify, and address, the sources of these systematic biases in data completeness.

2. **My second methodological issue is more basic. It seemed odd to me that the missing narratives were not discussed in the context of other missing variables. LE narratives, in particular, were probably missing due to a lack of law enforcement investigation (which you do mention). If the data allows, restricting your analysis to only cases where some other data from the same source was entered would be helpful. Otherwise, it is not clear if your results are just picking up on a lack of investigation, rather than a specific issue with the narratives themselves. Your second analysis, which focuses on the length of narratives, helped address this problem, but I think you need to be a bit more specific in either addressing why you did not filter out cases without ANY LE information, or remove them. I suspect that there is a strong correlation--the NVDRS training strongly discourages abstractors from skipping the narrative if any LE or CME information exists. If that is true, your paper may need to more clearly acknowledge that the missing narrative problem is directly and solely driven by missing data problem. If the correlation is moderate, you could include other variable missingness (as a percent, maybe?) as a variable in your regressions.**

Thank you for this comment. Generally, there is reatlively little missing data on the predictors/covariates used in the analysis as they are largely drawn from death certificates, as shown in Table 1 (i.e., only 89 observations (0.04%) in the dataset in total (that is, not conditional on having a narrative) were missing the variable “sex”). In addition, we explicitly model any covariate missingness in our regression models (see Supplemental Tables 1 and 2) - that is, we do not exclude any cases because of missingness on covariates, but instead include a dummy-coded “missing” value for every predictor in our analysis.

Finally, the analysis of narrative length (quasi-Poisson modeling) uses a truncated distribution of narratives to examine length, wherein we removed any observation with missing narratives from this analysis (either truly missing (character length=0) or assigned by us as being missing (character length<31, a threshold we determined through exploring the content of texts across different lengths).

In response to this comment, we have clarified how we handled missing data on covariates in the Methods (reprinted below):

“While the amount of missing data in these predictor variables was generally small (see Table 1a), we included a dummy code for missingness for all predictors so that these observations were retained in the regression analyses.”

**Reviewer #2 Comments to the Author**

1. **The authors use the length of the narrative (in terms of character count) as a proxy for the information potential and quality of text. There are limitations to this approach, as noted by the authors on page 25. It would be a useful addition to include a small random sample of narratives of different lengths to contextualize the differences in the quality of information contained in these narratives. Were any sensitivity analyses conducted to determine if the results differ if number of words (rather than number of characters including spaces) is used to proxy length?**

In response to this comment, we have added annotated examples of a random sample of five short (31 to <200 characters) and five long (>500 characters) C/ME and LE narratives to the **Appendix** (which also provides examples of texts that have <31 characters). The annotation describes the elements and features of these texts and serves as a crude proxy of the “information potential” of the texts.

We appreciate and understand the sentiment of this comment, but feel that a meaningful analysis of the information potential of these narrative texts is beyond the scope of this paper and would benefit from a data science (e.g., natural language processing, topic modeling) approach. We have added emphasis on this in the Discussion (please see revised text).

2. **P. 13: Given the possibility for coder bias, can the authors control for individual NVDRS coders and/or their length of experience? For example, if newly-added states to the NVDRS are more demographically diverse and less experienced coders are working on those narratives, it could skew results. I don’t know whether that’s the case but it’s one of several possibilities.**

Thank you for this comment. We do not have data on individual coders/NVDRS staff, unfortunately. However, our new analytic approach of accounting for clustering within states using Generalized Estimating Equations (GEE, see response to Reviewer #1) should account for factors like coder experience that cluster within site). Even with this new analytic approach, we still observe substantial disparities in narrative length by race/ethnicity in these data.

3. **There are also important differences in the background of medical examiners and coroners which may affect the original reports. The study controls for states, thus capturing potential state differences but beyond state controls, have they considered other ways to capture geographic differences in death investigation systems. E.g. https://www.cdc.gov/phlp/publications/coroner/death.html. Data on county of residence of the decedent are included in the NVDRS.**

Thank you for this comment. We believe our new modeling approach (GEE) is an appropriate means of addressing factors (like centralized vs. decentralized death examination systems) that vary across states.

We also want to clarify that our intent in this analysis is to *account for* state (NVDRS site) clustering as an *analytic issue*, not to identify the reasons for that state clustering (which is a distinct research question). We want to characterize the NVDRS data archive as a whole as a means to inform future research. Therefore, we do not feel it is appropriate to examine sub-site factors (e.g., county of death) in this analysis.

Please also see our response to comment #6 below.

4. **The authors might consider putting Tables 1 and 2 in the appendix and the regression analyses currently in the appendix in the body of the paper. Tables 1 and 2 should indicate whether the differences (e.g. between non-missing/missing and between C/ME and LE) are statistically significant.**

The findings shown in Supplemental Tables 2 and 3 (regression tables) are identical to Figures 2 and 3 in the main text, and therefore we think including them in the main text would be redundant. In contrast, current Tables 1 and 2 provide a description of the narrative data in absolute terms (vs. the regression tables which only show relative differences) and therefore we feel they provide valuable information for the reader that is not present elsewhere in the Results. As such, we have not made this suggested change.

We have elected not to include p-values in Table 1 because the purpose of this table is to describe the sample, rather than to test any particular hypotheses, and it contains a lot of information already. The Supplemental Tables provide the 95% confidence intervals for all these comparisons while accounting for state clustering.

5. **Regarding a dose response relationship between age and odds of a missing narrative “consistent for C/ME and LE texts” (p. 20, lines 244-246). According to the CI in Table 1, the effect of age on the C/ME missing is generally not significant, and there are no differences across the age groups in the effect. The patterns are different for LE missing.**

Thank you for this comment. We have now corrected the text to reflect that the relationship between age and narrative missingness is limited to LE texts.

6. **As alluded to in #2, the controls are interesting in their own right. For example, there are significant differences across states and over time in the patterns of missingness and narrative length. At a minimum, it would be useful to provide more discussion and suggestions for future research as to why these differences exist. Some of the between-state differences may relate to the points raised on page 24 (some could be tested explicitly – e.g. time in system).**

While we agree with the sentiment of the comment, our goal is to draw attention to these patterns to external researchers, like ourselves, who are interested in addressing substantive scientific questions with this archive can do so in a manner that accounts for the information bias we have identified.

In response to this comment we have added the ICCs that show the amount of clustering by state (see response to Reviewer #1, comment #1) and added language to the Discussion regarding suggestions for future research and collaborations between data creators and data users (see revised text, last paragraph of the Discussion).

7. **The discussion would also benefit from further explication of possible reasons as to why these patterns exist. For examples, studies of the Census undercount and/or inaccuracies in other official records would provide insight. The authors mention this only in the last sentence of the paper. It would be useful to synthesize and relate some of the possible explanations for these patterns in other sources to this analysis to provide a richer interpretation of the findings.**

While we agree with the sentiment of the comment, our goal is to draw attention to these patterns to external researchers, like ourselves, who are interested in addressing substantive scientific questions with this archive can do so in a manner that accounts for the information bias we have identified.

Moreover, we do not feel that our analysis can test the reasons for the patterns we observe, as we note in our discussion of study limitations, and that any comments we make as to their source would be speculative. That is, with the data we have we cannot capture important NVDRS system factors like updates to the data abstraction dashboard (i.e., introducing new variables, changing the coding of variables), changes in training, changes in staffing, new initiatives prioritizing data collection on certain groups, etc. that may contribute to these patterns. Other stakeholders (individual sites, the CDC) likely have data on these elements that likely could address these questions. Therefore, we feel collaboration with CDC and state NVDRS staff is the most effective means of identifying the reasons for these patterns - and addressing them in the future.

In response to this comment, we have added additional text to the Discussion on the need for collaboration between data creators and data users in the NVDRS to maximize the scientific utility of this archive. Please see the final paragraph of the Discussion.
