# Supplementary material for: Not discussed: Inequalities in narrative text data for suicide deaths in the National Violent Death Reporting System
Source: PLoS One. 2021 Jul 16;16(7):e0254417. doi: 10.1371/journal.pone.0254417 (PMC8284808; doi:10.1371/journal.pone.0254417)
Supplement: Attachment — Submitted filename: Response to Reviewers.docx [file pone.0254417.s011.docx]

**COMMENTS FROM EDITOR AND REVIEWERS**

**Comments from the Editor:**

Both of the reviewers and I agree that most of the issues raised in the first round of reviews have been well-addressed, and that the manuscript is much improved. Each reviewer however raises one or more remaining but minor issues that would further improve the paper. Reviewer 1 would like to have a fuller disclosure of the data sources to which you did or did not have access -- this would be a very helpful step for future research in the area. Reviewer 2 recommends addressing the missingness of the decedent characteristic of education, since it is higher than the other characteristics, and suggests a sensitivity analysis excluding deaths of undetermined cause. Please either make these changes or explain why you are not doing so.

Overall, however the paper makes a strong contribution, and the Appendix with text examples is particularly enlightening.

**Response**: Thank you.

As outlined in the response letter, we have conducted three additional sensitivity analyses (1. Imputing education level, 2. Excluding cases of undetermined death, and 3. Additionally adjusting for presence of a toxicology report) to respond to these remaining comments, the results of which are shown in new **Supplemental Tables 5-8**. While these tables show that our substantive conclusions are unaffected by these factors, we feel that these additional analyses strengthen the overall contribution to the paper by excluding these factors as driving our findings.

Regarding Reviewer #1, we have now added additional language about the variables that may be in the NVDRS that are not available to outside researchers like ourselves; we also emphasize that our main analysis does account for many system-level ‘meta-variables’ that Reviewer #1 references, such as “circumstances known” as well as state and year.

We hope you will now find our manuscript acceptable for publication. We look forward to hearing from you.

**Reviewers' comments:**

**Reviewer #1:** This version represents a substantial improvement over the original, and addresses all of my major concerns with the first version. I have only one lingering concern.

1. **While I understand and support the authors' decision to not speculate on the mechanisms behind narrative length, I still think it needs to be more explicitly stated, earlier than in the discussion, that narratives are generally missing because the underlying document/information needed to complete a narrative was missing--and that the presence of these documents can be ascertained in other ways. In my initial review, I assumed the authors had access to the LE/CME specific variables that the NVDRS collects (such as toxicology, death circumstances, etc), which would be an easy way to check the assumption that a lack of a narrative implies a lack of (access to) a CME/LE, but the writing suggests that those are not available to the authors. I thought they were available in the restricted-access version of the NVDRS data. They are correct that death certificates do not provide the needed information--in our state, even the "autopsy performed" variable does not necessarily reflect whether or not our team had access to an autopsy. If it is not possible within the scope of the authors' data access to adjust for the presence of CME/LE data, it should at least be noted that the data exists, and could be utilized by someone with different permissions or working with a different time period. If the authors did have access to these variables and chose not to use them, a more robust explanation of the rationale is needed. I think it would be appropriate to consider these variables even if they do not exist for portions of the study period (as NVDRS data collection does change frequently, as the authors noted).**

Thank you for this comment. We want to reiterate that we do control for several system-level variables such as “circumstances_known,” (indeed, the narrative length analysis is conditional on this variable being coded “yes”), “death place,” “autopsy,” “state,” and “year,” in all our analysis, in order to isolate the relationship between the demographic predictors and narrative length from these potential confounding variables.

We have edited the manuscript in several ways to address this concern:

(1) In the Methods section (*Qualitative text narratives*) we have expanded this section to include the italicized text to emphasize the importance of the source materials as a determinant of the narrative missingness and length (added text in italics): “If the patterns are similar, this may reflect features of the centralized NVDRS system or general limitations in the accuracy and completeness of mortality documentation *(i.e., lack of access to specific records by NVDRS staff,* incomplete death certificates) [26]. If the patterns differ, this may reflect characteristics of the source documents *(e.g., toxicology reports, police reports)* or reporting procedures.”

(2) We have conducted an additional sensitivity analysis adjusting for the presence of a toxicology report (coded as Yes vs. No/Not Applicable by the NVDRS). The results of this analysis are presented in Model 4 of new Supplemental Tables 5-8. This new analysis shows that decedents who had a toxicology report had significantly shorter CME narratives (Supplemental Table 7, Model 4: IRR=0.90) but that having a toxicology report was not associated with length of LE narratives (Supplemental Table 8, Model 4: IRR: 0.99); as a reminder, the analysis of narrative length is limited to those observations in which the NVDRS coder indicated that “death circumstances” were “known” AND that the narrative has a length of at least 31 characters.

Most importantly, as shown by these results, even after accounting for circumstances known, whether an autopsy was performed, toxicology report, and having a minimum narrative length, the substantive findings from our analysis are unchanged: decedents who are older, are male, are Black/African American or Asian/Pacific Islander, and who have less education have significantly shorter narratives (both CME and LE).

(3) Finally, we have added new text to the Discussion about the variables in the NVDRS system that may exist but are not available as part of the RAD that could be used by internal NVDRS staff to identify the specific reasons why a particular narrative is missing (e.g., flags indicating that the case incident report was incomplete/needed follow-up; the source that the NVDRS staff requested the document from; whether or not the document was available to the coder), but which are not part of the data that external researchers can request.

This is reprinted here: “Finally, while not part of the RAD that external researchers can access, there may be data processing variables that are created as part of the NVDRS abstraction process that internal staff could use to identify the specific reasons why a particular narrative is missing (e.g., indicators that the incident report needed follow-up; the specific document source; whether or not the document was available to the coder), which the CDC could use to identify system-level factors that contribute to data (in)completeness.”

2. **Some information on the correlation between missing source data and narrative length would be so helpful.**

This information in Supplemental Tables 7 and 8, which show the relationship between all values of the predictor variables (including the dummy code for missing data) and narrative length for CME (Supplemental Table 7, Model 1) and LE (Supplemental Table 8, Model 1) narratives.

As these tables show, in general there is not a consistent pattern between being missing on a predictor variable and narrative length; missing education is associated with slightly longer LE narratives (IRR=1.03) but slightly shorter CME narratives (IRR=0.97), for example.

3. **I think that these concerns could be addressed briefly in the text. The discussion surrounding state variation was great, and already touches on some of the reasons why abstractors may not have access to these documents, so it shouldn't take much tweaking to address the other variables. Either way, I think the narratives represent a better aggregate measure of data availability than any one variable--the discussion surrounding narrative length was particularly interesting.**

Thank you for this, and please see our responses to items 1 and 2 above.

4. **I also agree with Reviewer 2 and wish that the regression tables were available in the main text. I find them easier to interpret than the forest plots. However, that's more of a personal preference than a true problem with the paper.**

We prefer to present the figures in the main text and the tables in the supplement. Thank you.

**Reviewer #2:** The authors have done a good job of addressing concerns raised in the first set of reviews. I have just a couple of additional points of clarification:

**1. Although the level of missingness for decedent characteristics other than the presence of a narrative is generally low, that is not the case for education (25-30% of cases lack this information). Given this high level of missingness and the fact that including a dummy variable to indicate missing status can lead to biased estimates (Paul Allison and others), I recommend that the authors use multiple imputation instead.**

Thank you for this comment. We agree, and in response we conducted an additional sensitivity analysis with imputing education using multivariate chained equations (30 datasets across 20 iterations) (see revised Methods).

Findings for the outcome of missing narrative are shown by Model 2 in new Supplemental Tables 5 (CME) and 6 (LE); findings for the outcome of narrative length are shown by Model 2 in Supplemental Tables 7 (CME) and 8 (LE). As shown by these results, are findings are largely unchanged using the imputed education variable, even if not every point estimate is statistically significant: higher education is inversely associated with the narrative being missing, particularly for the CME narratives, and, conditional on having a non-missing narrative, higher education is associated with longer texts for both CME and LE narratives. We have now summarized these findings in the final paragraph of the Results (see revised text).

**2. Did the authors conduct a sensitivity analysis to determine whether the substantive conclusions are changed if the deaths of undetermined cause are excluded? This would be a worthwhile check, and results could simply be reported in the text.**

In response to this comment we re-ran all our analyses excluding undetermined deaths. These results are shown by Model 3 in new Supplemental Tables 5 and 6 (missing CME and LE narratives, respectively) and Model 3 in new Supplemental Tables 7 and 8 (length of CME and E narratives, respectively). The results show that our findings are substantially unchanged from our main analysis (shown in Model 1 in these Supplemental Tables). The findings are summarized in the text (reprinted below): “The results from sensitivity analysis excluding all cases of undetermined cause of death were substantially unchanged from our main analysis.”
